# Dissecting Causal Associations of Diet-Derived Circulating Antioxidants with Six Major Mental Disorders: A Mendelian Randomization Study

**DOI:** 10.3390/antiox12010162

**Published:** 2023-01-10

**Authors:** Hao Zhao, Xue Han, Xuening Zhang, Lingjiang Li, Yanzhi Li, Wanxin Wang, Roger S. McIntyre, Kayla M. Teopiz, Lan Guo, Ciyong Lu

**Affiliations:** 1 Department of Medical Statistics and Epidemiology, School of Public Health, Sun Yat-sen University, Guangzhou 510080, China; 2 Guangdong Provincial Key Laboratory of Food, Nutrition and Health, Sun Yat-sen University, Guangzhou 510080, China; 3 Department of Psychiatry, Shenzhen Nanshan Center for Chronic Disease Control, Shenzhen 518054, China; 4 Department of Epidemiology and Biostatistics, College of Public Health, Shandong University, Jinan 250012, China; 5 Mental Health Institute of the Second Xiangya Hospital, Central South University, Changsha 410011, China; 6 Department of Psychiatry, University of Toronto, Toronto, ON M5T 1R8, Canada; 7 Department of Pharmacology, University of Toronto, Toronto, ON M5S 1A8, Canada; 8 Institute of Medical Science, University of Toronto, Toronto, ON M5S 1A8, Canada; 9 Mood Disorders Psychopharmacology Unit, University Health Network, Toronto, ON M5T 2S8, Canada

**Keywords:** antioxidant, mental disorders, Mendelian randomization, genome-wide association studies, oxidative stress

## Abstract

Although observational studies have suggested associations between circulating antioxidants and many mental disorders, causal inferences have not been confirmed. Mendelian randomization (MR) analyses were conducted using summary-level statistics from genome-wide association studies (GWASs) to explore whether genetically determined absolute circulating antioxidants (i.e., ascorbate, retinol, β-carotene, and lycopene) and metabolites (i.e., α- and γ-tocopherol, ascorbate, and retinol) were causally associated with the risk of six major mental disorders, including anxiety disorders (AD), major depressive disorder (MDD), bipolar disorder (BIP), schizophrenia (SCZ), post-traumatic stress disorder (PTSD), and obsessive–compulsive disorder (OCD). MR analyses were performed per specific-outcome databases, including the largest GWAS published to date (from 9725 for OCD to 413,466 for BIP participants), UK Biobank (over 370,000 participants), and FinnGen (over 270,000 participants), followed by meta-analyses. We found no significant evidence that genetically determined diet-derived circulating antioxidants were significantly causally associated with the risk of the six above-mentioned major mental disorders. For absolute antioxidant levels, the odds ratios (ORs) ranged from 0.91 (95% CI, 0.67–1.23) for the effect of β-carotene on OCD to 1.18 (95% CI, 0.90–1.54) for the effect of ascorbate on OCD. Similarly, for antioxidant metabolites, ORs ranged from 0.87 (95% CI, 0.55–1.38) for the effect of ascorbate on MDD to 1.08 (95% CI, 0.88–1.33) for the effect of ascorbate on OCD. Our study does not support significant causal associations of genetically determined diet-derived circulating antioxidants with the risk of major mental disorders.

## 1. Introduction

According to the latest data on the global burden of disease, from 1990 to 2019, the disability-adjusted life years caused by mental disorders increased from 80.8 million to 125.3 million, and the percentage contributed by mental disorders increased from 3.1% to 4.9% [1]. Moreover, due to the COVID-19 pandemic, people are facing unprecedented levels of established mental health risk factors, including social isolation [2], stress [3], and anticipated economic hardship [4], and the issue of mental disorders is amplified, with increasing public health concerns [5]. Anxiety disorders (AD), major depressive disorder (MDD), bipolar disorder (BIP), schizophrenia (SCZ), post-traumatic stress disorder (PTSD), and obsessive–compulsive disorder (OCD) are common major mental disorders in adults, which overlap in genetic and clinical aspects [6,7], indicating that there may share etiological mechanisms. Although considerable efforts have been made to understand the nature of mental disorders, understandings of their pathogenesis remain limited, and there are no effective etiological prevention methods. Previous studies have reported that oxidative stress can cause oxidative damage to biological macromolecules, cells, and neurons, which is considered to be one of the primary pathogeneses of mental disorders [8]. Moreover, it has been reported that antioxidants, which can help eliminate free radicals and reduce and eliminate oxidative damage [9], would be potential targets for the primary prevention of mental disorders. In addition to the endogenous antioxidant enzyme systems, antioxidants from dietary intake are the most easily accessible and modifiable approach for consideration.

Current research on the relationship between antioxidants and mental disorders has mixed results. Some observational studies have shown that dietary intake, either as dietary components or supplements, or the concentration of vitamins E and C and carotenoids in the blood are associated with a reduced risk of AD [10], MDD [11,12,13], BIP [14], SCZ [15,16], PTSD [17], and OCD [18]. However, some studies have not reported the protective effects of the preceding antioxidants on mental disorders [19,20,21,22,23,24,25]. The inconsistent results reported in some observational studies may be due to uncertain temporal relationships, insufficient sample sizes, or potential confounding factors. Except for a few anxiety and depression prevention studies, most previous randomized clinical trials (RCTs) of antioxidants have focused more on improving symptoms in people with mental disorders [26,27], so there is still a lack of RCTs investigating whether diet-derived antioxidants can prevent mental disorders. However, intervention studies in healthy people cannot be conducted without sufficient evidence because of the potential for unknown risks and harm to the subjects. Moreover, intervention trials are often limited by timing, dosage, duration, use of natural or synthetic antioxidants, and the uncertainty of the onset time and long-term progression of mental disorders. Therefore, the causal relationship between diet-derived antioxidants and the risk of major mental disorders remains unclear.

Mendelian randomization (MR), which uses genetic variants of the exposure as instrumental variables to minimize measurement errors and confounding and reversed causation, can provide a reliable estimation of the causal association between exposure and outcomes under specific assumptions [28]. Although a previous study based on MR analysis found a potential causal association between several antioxidants from questionnaires (such as ascorbate and retinol) and psychiatric disorders [29], notably, antioxidant levels estimated from questionnaires might not accurately represent circulating antioxidant levels in the blood.

Therefore, in this study, we performed two-sample MR analyses to assess the causal associations between genetically determined diet-derived circulating antioxidant levels in the blood and six major mental disorders, including AD, MDD, BIP, SCZ, PTSD, and OCD.

## 2. Materials and Methods

### 2.1. Overall Study Design

**Figure 1:**
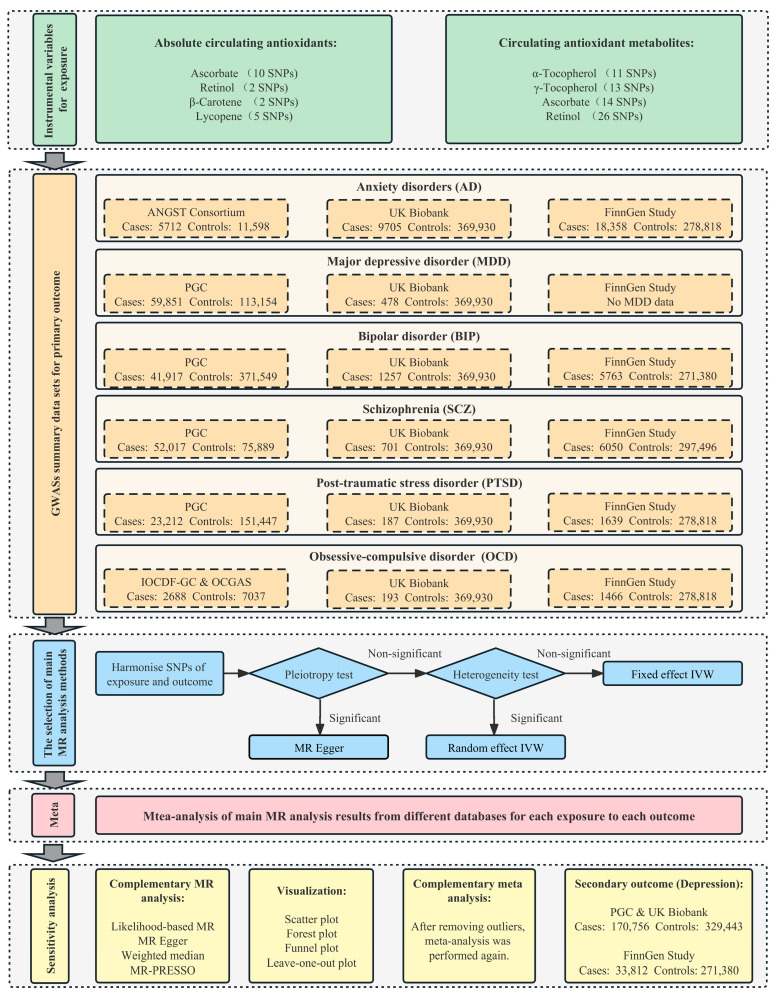
Schematic overview and framework of the present MR study design. Abbreviations: SNP, single-nucleotide polymorphism; MR, Mendelian randomization; PGC, Psychiatric Genomics Consortium; IVW, inverse-variance weighted; MR PRESSO, MR Pleiotropy RESidual Sum and Outlier.

The study herein used 2-sample MR analyses of summary statistics from genome-wide association studies (GWASs) to investigate whether diet-derived circulating antioxidants, including vitamins E (α- and γ-tocopherol), ascorbate, retinol, β-carotene, and lycopene, were causally associated with the risk of six major mental disorders, including AD, MDD, BIP, SCZ, PTSD, and OCD. We considered the following two phenotypes for these antioxidants as exposure: (1) absolute circulating antioxidants measured as authentic absolute levels in the blood, and (2) circulating antioxidant metabolites quantified as relative concentrations in plasma or serum, either or both. The instrumental variables needed to satisfy three assumptions: the relevance assumption, the independence assumption, and the exclusion restriction assumption [30]. For each mental disorder as an outcome, MR analyses were performed in each of three European databases (the largest GWAS published to date, UK Biobank, and the FinnGen study), and these were subsequently meta-analyzed to approximate the average genetically influenced effect on each specific outcome. This study was reported according to the Strengthening the Reporting of Observational Studies in Epidemiology Using Mendelian Randomization (STROBE-MR) checklist. The schematic overview and framework of the present study design are shown in Figure 1.

### 2.2. Determination of Exposures

For instrumental variables on absolute circulating antioxidants, genetically determined α-tocopherol, ascorbate, retinol, and β-carotene were identified in the recent large-scale GWAS (*p* < 5 × 10^−8^, linkage disequilibrium [LD]: r^2^ = 0.001 and clump distance = 10,000 kb). Three independent single nucleotide polymorphisms (SNPs) associated with α-tocopherol were identified from a GWAS on 4014 individuals of European ancestry [31]; however, these three SNPs were reported to be related to lipid metabolism or regulation and therefore were not considered for MR analysis due to possible pleiotropic bias. Eleven SNPs associated with ascorbate were identified from a recently published GWAS on 52,018 individuals of European ancestry [32]. One (rs7740812) of the 11 SNPs was removed for subsequent MR analysis due to the LD with other variants (r^2^ > 0.001) or absence from the LD reference panel using the EUR population reference. Two independent SNPs associated with retinol were identified from a GWAS of 5006 Caucasian individuals in two cohort studies [33]. Two independent SNPs associated with β-carotene were identified from a GWAS of 2344 participants in the Nurses’ Health Study [34]. Five independent SNPs associated with lycopene were identified from a GWAS on 441 Caucasian participants under the relaxed threshold criteria (*p* < 5 × 10^−6^) [35].

For instrumental variables on circulating antioxidant metabolites, genetically determined α-tocopherol, γ-tocopherol, ascorbate, and retinol were extracted from the metabolite GWAS analysis under the relaxed threshold criteria (*p* < 1 × 10^−5^). In total, 11 SNPs for α-tocopherol, 13 SNPs for γ-tocopherol, and 14 SNPs for ascorbate were derived from two European population studies [36] and 26 SNPs for retinol from 1960 adults of European descent [37]. 

Based on PhenoScanner, the SNPs that did not reach a significant association with the confounders were retained in the filtered genetic instrument [38]. The F statistic for each SNP was calculated by the formula Beta^2^/SE^2^, and the F statistics of >10 for each SNP was recommended for subsequent MR analysis to avoid employing weak genetic instruments.

### 2.3. Data Sources of Major Mental Disorders

Summary-level statistics for each mental disorder were obtained from three large databases, including the largest GWAS published by European-ancestry to date, UK Biobank, and the FinnGen study. Neither the UK Biobank nor the FinnGen study were major parts of the largest GWAS study, preventing the inclusion of overlapping samples. For SNPs of the instrument that were not available in the outcome GWAS, the LDlink tool was used to identify proxy SNPs of European ancestry [39]. SNPs missing in the outcome GWAS without appropriate proxy SNPs available were then excluded.

The largest GWAS summary statistics for AD, MDD, BIP, SCZ, PTSD, and OCD were extracted from the Psychiatric Genomics Consortium (PGC) website (https://www.med.unc.edu/pgc/results-and-downloads/ (accessed on 25 June 2022)). The PGC is the largest consortium in the history of psychiatry, which has conducted the most influential meta- and mega-analysis of genome-wide genomic data for mental disorders. The GWAS summary datasets of European-ancestry, for AD (5712 cases and 11,598 controls) from the Anxiety NeuroGenetics STudy (ANGST) Consortium in 2016 [40], MDD (59,851 cases and 113,154 controls) from PGC in 2018 [41], BIP (41,917 cases and 371,549 controls) from PGC in 2021 [42], SCZ (52,017 cases and 75,889 controls) from PGC in 2022 [43], PTSD (23,212 cases and 151,447 controls) from PGC in 2019 [44], and OCD (2688 cases and 7037 controls) from the International Obsessive–Compulsive Disorder Foundation Genetics Collaborative (IOCDF-GC) and the OCD Collaborative Genetics Association Study (OCGAS) in 2017 [45] were obtained from genome-wide meta- or mega-analyses.

The UK Biobank was a population-based prospective cohort study with deep genetic, physical, and health data collected on about 500,000 individuals, aged 40–69 years, across the UK from 2006 to 2010. The second-round analysis of UK Biobank data from the Pan-UK Biobank project (https://pan.ukbb.broadinstitute.org/ (accessed on 25 June 2022)) was used in the present study. Based on the code, there were 9705 cases of AD, 478 cases of MDD, 1257 cases of BIP, 701 cases of SCZ, 187 cases of PTSD, 193 cases of OCD, and 369,930 controls. The FinnGen summary statistics were from release 7 in 2022 (https://finngen.gitbook.io/documentation/data-download (accessed on 25 June 2022)). There were 18,358 cases of AD, 5763 cases of BIP, 6050 cases of SCZ, 1639 cases of PTSD, 1466 cases of OCD, and over 270,000 controls.

### 2.4. Statistical Analysis

The selection process of the main MR analysis methods is shown in Figure 1. When the MR-Egger intercept test found that there may be potential horizontal pleiotropy, MR-Egger regression was performed with pleiotropy-robust causal estimates by bootstrapped standard errors [46]. Notably, the existence of significant horizontal pleiotropy was extremely rare due to the rigorous screening of instrumental variables. The inverse-variance weighted (IVW) meta-analysis assumes that either all the instruments are valid or any horizontal pleiotropy is balanced. Heterogeneity was detected using Cochran’s Q-statistics test. If there was significant heterogeneity, the random-effect IVW model was used as the main analysis; otherwise, the fixed-effect IVW model was used as the main analysis [47].

For each specific exposure and each specific outcome, MR analyses were performed separately in each of the specific-outcome databases from different sources and then were subsequently meta-analyzed to generate the pooled estimates for each specific exposure on the risk of each specific outcome. Through Cochran’s Q-statistics test, we calculated I^2^ statistics to quantify heterogeneity between estimates from different databases and corresponding *p* values. If there was no significant heterogeneity across different databases, fixed-effect model meta-analyses were used to pool estimates across the databases from different sources for each specific exposure. If there was significant heterogeneity after excluding the obvious clinical heterogeneity, the random effect model meta-analyses were used to pool estimates.

### 2.5. Sensitivity Analysis

To further assess the robustness of our findings, a series of sensitivity analyses was performed. First, complementary MR analyses with different assumptions were applied to help verify causal inference. The likelihood-based MR can effectively estimate the log-linear association between exposure and outcome risk (No. SNPs > 1) [48]. The weighted median can provide valid estimates if at least 50% of the weight comes from valid instrumental variables (No. SNPs > 2) [49]. The MR Pleiotropy RESidual Sum and Outlier (MR-PRESSO) was performed, which detects and corrects the effects from outliers (No. SNPs > 3) [50]. Second, we also performed scatter, forest, funnel, and leave-one-out plots to detect high influence points. Third, if MR-PRESSO detected outliers, the meta-analysis was performed again for each specific exposure and each specific outcome by removing the outliers. Finally, as only depression and no MDD phenotypes were found in the FinnGen database, we considered depression as a secondary outcome and estimated the effect of circulating antioxidants on broader depression through MR analysis in two GWAS analyses, the PGC and UK Biobank study (170,756 cases and 329,443 controls) [51], and the FinnGen study (33,812 cases and 271,380 controls).

The prior statistical power was calculated using the mRnd power calculation online tool [52]. Given a type 1 error of 5%, we had sufficient power (>80%) to detect the minimum detectable OR. To account for multiple testing in our analyses, we used a Bonferroni-corrected threshold of *p* < 0.001 (α = 0.05/48) as significant evidence of associations, and a *p*-value between 0.05 and 0.001 was considered suggestive evidence of associations. All statistical analyses were performed using R version 4.1.0.

## 3. Results

### 3.1. Strength of Genetic Instruments

The summary information on instrumental variables for absolute circulating antioxidants and antioxidant metabolites is presented in Table 1. The information on the cohorts contributing to the GWAS of absolute antioxidant levels is given in Table S1. In our study, the F statistic of each SNP was greater than 10, indicating that the instrumental variables of antioxidants could better avoid the bias of potentially weak instrumental variables. The summary information of GWAS for six major mental disorders is shown in Table S2. Although MDD was not found in the FinnGen study, the other five outcomes used three GWAS databases from different sources. As shown in Table S3, the minimum detectable effect sizes were reasonable at sufficient power, especially for the largest GWAS source databases. Therefore, most of our studies had enough power given the current parameters. The raw data information on the effect estimation for the associations of selected SNPs with antioxidants and with major mental disorders is given in Tables S4 and S5.

### 3.2. Effect of Absolute Circulating Antioxidants on the Risk of Major Mental Disorders

**Figure 2:**
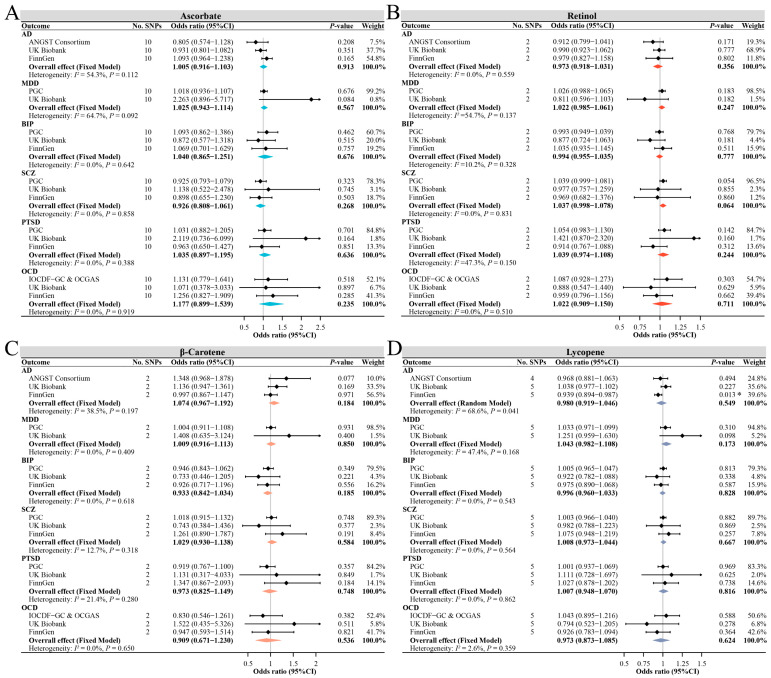
The main MR analysis results of the causal effects of four absolute circulating antioxidant levels on six major mental disorders. The ORs are scaled per µmol/L increase in ascorbate (**A**), per 0.1 unit increase in ln-transformed retinol (**B**), per unit increase in ln-transformed β-carotene (**C**), and per µg/dL increase in lycopene (**D**). The asterisk represents a suggestive association (0.001 < *p*-value < 0.05). Abbreviations: AD, anxiety disorders; MDD, major depressive disorder; BIP, bipolar disorder; SCZ, schizophrenia; PTSD, post-traumatic stress disorder; OCD, obsessive–compulsive disorder.

The primary results of the MR estimate for absolute circulating antioxidants are presented in Figure 2. For instrumental variables with three or more SNPs, the MR-Egger intercept test found no significant horizontal pleiotropy for all outcomes; therefore, the IVW method was used as the primary analysis results. Genetically determined absolute ascorbate levels were not associated with the risk of each mental disorder in any database, with the pooled ORs per 1 µmol/L of ascorbate ranging from 0.93 (95% CI, 0.81–1.06) for SCZ to 1.18 (95% CI, 0.90–1.54) for OCD. Genetically determined absolute retinol levels were also not associated with the risk of each mental disorder in any database, with the pooled ORs per 0.1 ln-transformed retinol ranging from 0.97 (95% CI, 0.92–1.03) for AD to 1.04 (95% CI, 0.97–1.11) for PTSD. Similarly, genetically determined absolute β-carotene levels were not associated with the risk of each mental disorder in any database, with the pooled ORs per ln-transformed β-carotene ranging from 0.91 (95% CI, 0.67–1.23) for OCD to 1.07 (95% CI, 0.97–1.19) for AD. Except for the suggestive association of absolute lycopene with the risk of AD found only in the FinnGen study, we observed no evidence that absolute lycopene levels were associated with the risk of each mental disorder. Notably, the pooled estimates were not significantly associated between lycopene and the risk of any mental disorder, with the pooled ORs per 1 µg/dL of lycopene ranging from 0.97 (95% CI, 0.87–1.09) for OCD to 1.04 (95% CI, 0.98–1.11) for MDD.

The supplementary MR analyses showed that the results of the likelihood-based MR method were in good agreement with the IVW results, and the results of the MR-Egger and weighted median method for ascorbate and lycopene were also comparable to the IVW results, even though their estimates were more conservative (Tables S6–S9). The MR-PRESSO method identified one outlier SNP for ascorbate on BIP in PGC and FinnGen and for ascorbate on SCZ in PGC. MR analyses after removing this outlier showed that the OR in the corresponding database and combined OR estimates did not change significantly (Figure S1). No outlier SNPs were identified in the MR-PRESSO analysis for the other outcomes, and the leave-one-out analyses also found no significant outliers. The combined estimates also found no significant causal association between absolute antioxidant levels and depression (as a secondary outcome), which was consistent with the results of MDD (Figure S2).

### 3.3. Effect of Circulating Antioxidant Metabolites on the Risk of Major Mental Disorders

**Figure 3:**
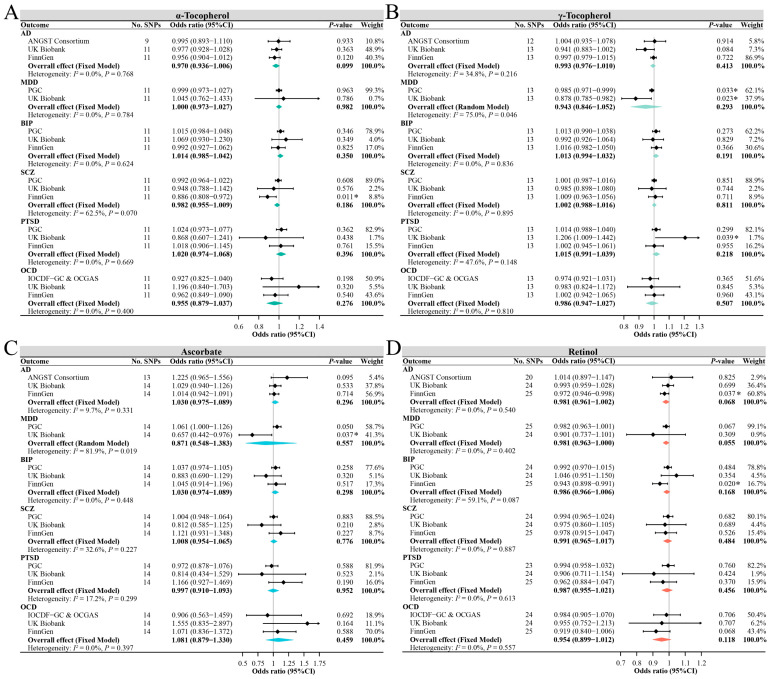
The main MR analysis results of the causal effects of four circulating antioxidant metabolites on six major mental disorders. The ORs are scaled per 0.1 unit increase in log-transformed α-tocopherol (**A**) and γ-tocopherol (**B**) and per unit increase in log-transformed ascorbate (**C**) and retinol (**D**). The asterisk represents a suggestive association (0.001 < *p*-value < 0.05). Abbreviations: AD, anxiety disorders; MDD, major depressive disorder; BIP, bipolar disorder; SCZ, schizophrenia; PTSD, post-traumatic stress disorder; OCD, obsessive–compulsive disorder.

Figure 3 shows the primary results of MR estimates for circulating antioxidant metabolites. We used the IVW method as the primary analysis outcome, except γ-tocopherol for AD in the UK Biobank database and ascorbate for OCD in the IOCDF-GC and OCGAS database, using the MR-Egger method due to possible potential pleiotropy (Tables S10–S13). Although some suggestive associations were found in some single databases, such as α-tocopherol for SCZ in the FinnGen database, γ-tocopherol for MDD in the PGC and UK Biobank databases, γ-tocopherol for PTSD in the UK Biobank database, ascorbate for MDD in the UK Biobank database, and retinol for AD and BIP in the FinnGen database, the combined estimates showed no significant causal association between any of the four antioxidant metabolites and the risk of any of the six mental disorders. For per 0.1 increase in log-transformed α-tocopherol, the pooled ORs ranged from 0.96 (95% CI, 0.88–1.04) for OCD to 1.02 (95% CI, 0.97–1.07) for PTSD. Similarly, for per 0.1 increase in log-transformed γ-tocopherol, the pooled ORs ranged from 0.94 (95% CI, 0.85–1.05) for MDD to 1.02 (95% CI, 0.99–1.04) for PTSD. For per increase in log-transformed ascorbate, the pooled ORs ranged from 0.87 (95% CI, 0.55–1.38) for MDD to 1.08 (95% CI, 0.88–1.33) for OCD. For per increase in log-transformed retinol, the pooled ORs ranged from 0.95 (95% CI, 0.90–1.01) for OCD to 0.99 (95% CI, 0.97–1.02) for SCZ.

Similarly, the supplementary MR analyses showed that the results obtained by the likelihood ratio method, weighted median method, MR-Egger method, and IVW method were comparable (Tables S10–S13). The MR-PRESSO method identified 1 outlier SNP for α-tocopherol on AD in FinnGen, for γ-tocopherol on AD in PGC, and for retinol on AD in PGC. MR analyses after removing this outlier showed that the OR in the corresponding database and combined OR estimates did also not change significantly (Figure S3). No outlier SNPs were identified in the MR-PRESSO analysis for the other outcomes, and the leave-one-out analyses also found no significant outliers. The combined estimates also found no significant causal association between antioxidant metabolites and depression (as a secondary outcome), which was consistent with the results of MDD (Figure S4).

## 4. Discussion

This study investigated the causal associations between diet-derived circulating antioxidants and the risk of six major mental disorders based on MR analyses. The genetic variation of circulating antioxidants was evaluated as authentic absolute blood levels and metabolite concentrations as instrumental variables, and comparable results were obtained. Although several suggestive associations were found in a single database, these were considered to be chance findings because no significant causal association between any antioxidants and any mental disorders was found in the combined database estimates. Therefore, we found no evidence to support a significant causal relationship between genetically determined diet-derived antioxidants and the risk of six major mental disorders.

Two retrospective reviews of the role of vitamin C in human mental disorders found current clinical evidence to be limited and inconclusive [53,54]. Two systematic reviews involving antioxidant nutritional supplements for depression and bipolar disorder found no significant effect of vitamin C on reducing depressive symptoms and preventing or treating bipolar disorder [23,55]. The World Federation of Societies of Biological Psychiatry guidelines also reported no evidence to support the treatment of mental disorders with antioxidants such as vitamin C [56]. A study based on the National Health and Nutrition Examination Survey also found no differences in the intake of antioxidants, including vitamins A, C, and E, between patients with SCZ and healthy controls [24]. These results are consistent with our findings. However, a meta-analysis of the effects of α-tocopherol on depression and anxiety showed that vitamin E supplementation could not improve anxiety but could improve depressive symptoms, but this meta-analysis had substantial heterogeneity (89–95%) due to the small sample size (less than 50 samples in half of the included studies), multiple interventions (mixed with components such as omega-3 fatty acids), and inconsistent outcome measurements [19], with the result being that this finding should be interpreted cautiously.

The current RCTs focus more on the role of antioxidants in the treatment of mental disorders, and there is a lack of large RCTs on whether the supplementation of antioxidants can reduce the risk of mental disorders. For example, in a study of Alzheimer’s disease, also a neurological disorder, an RCT of about 4000 people found no statistically significant effect of antioxidant supplementation on cognitive function [57], and a recent MR study of circulating antioxidants and Alzheimer’s disease also showed that higher levels of ascorbate, β-carotene, and retinol exposure did not reduce the risk of Alzheimer’s disease [58]. This also suggests that MR studies based on instrumental variables proxying circulating antioxidants from a genetic perspective have obtained comparable and consistent conclusions with empirical studies from large RCTs. Unfortunately, no MR studies have assessed the causal relationship between circulating antioxidants and the risk of major mental disorders to date. A previous MR study of circulating antioxidants has demonstrated that the effect of genetic variants on circulating antioxidant levels is generally comparable with those that would be achieved by dietary supplementation [59]. Considering the robust and broadly consistent null results in this study that neither absolute blood antioxidant levels nor metabolites measured by high-throughput techniques were causally associated with major mental disorders, dietary supplements that increase blood antioxidant concentrations may not reduce the risk of major mental disorders in healthy people.

Oxidative stress is an imbalance between oxidants and antioxidants in favor of the oxidants, leading to a disruption of redox signaling and control and/or molecular damage [60]. Our genetic findings do not contradict the hypothesis that oxidative stress plays an important role in the pathogenesis of mental disorders. The results may be related to the fact that the circulating antioxidant levels do not necessarily correspond to antioxidant nutritional intake [12]. Moreover, circulating levels of vitamin E do not provide complete insight in its antioxidant capacity [61]. Therefore, for healthy adults without nutritional deficiency and pathological damage, genetic evidence does not support the significant protective effect of dietary supplements that increase the concentration of blood antioxidants on the prevention of mental disorders. In addition, mental disorders may be associated with oxidative stress, and even if genetically determined diet-derived circulating antioxidants are not significantly associated with the risk of mental disorders, patients may benefit from antioxidant supplementation to reduce damages due to mental disorders. In the future, more real-world data on oxidative stress assessment will be needed to guide public health and clinical practice in mental disorders.

There are three main strengths in the present study. First, the MR design of two independent samples based on instrumental variables reduces the possibility of subjects being exposed to unnecessary risks and hazards in clinical trials and expands the genetic theoretical basis of dietary antioxidants and major mental disorders. Second, we used two independent sets of genetic instrument variables as proxies for dietary-derived antioxidants for ascorbate and retinol, including the absolute circulating antioxidants and their metabolites. Third, for each outcome except MDD, we conducted a separate analysis in three databases followed by a meta-analysis, which added to the robustness of our results.

However, some limitations should also be considered concerning the interpretation of the results. First, based on published summary data, we were unable to test nonlinear causal relationships between antioxidant levels and risk for select mental disorders. Second, the number of SNPs currently identified as instrumental variables for antioxidants is limited, but their ideal representation has been widely used in previous MR studies [59,62]. Also limited by the number of SNPs of instrumental variables, the MR-Egger, weighted median, and MR-PRESSO methods could not be performed for absolute retinol and β-carotene [33,34], but these instruments are mapped in genes critical to antioxidant metabolism and are not associated with any other risk factors for mental disorders in the PhenoScanner database [38], indicating that horizontal pleiotropy is unlikely to exist. In addition, the SNPs related to β-carotene were obtained from the Nurses’ Health study in the female population. In the future, it will be necessary to further discover more relevant sites through more large GWASs of antioxidants to improve the instrument variable strength further. Third, due to the rarity of some people with a mental health condition in the UK Biobank or FinnGen databases, it is also evident from our power calculations that there is not enough power in the database with a low proportion of patients, which may explain several associations that are thought to be chance. However, importantly, our findings on mental disorders based on different analysis methods are robust, and the results of the largest GWAS and meta-analyses to date are generally consistent. Fourth, we found that the meta-GWAS analysis for ascorbate by Zheng et al. [32] showed a considerable variation in detection concentrations in different cohorts, and it is necessary to standardize homogeneous detection techniques in the future. Finally, we could not explore these associations in nutritionally deficient populations that might be more promising with antioxidant supplementation or to test the effects of antioxidants in combination with other treatments. Furthermore, due to the availability of data, this study focused on populations of European ancestry, and the associations in other populations need further validation.

## 5. Conclusions

In summary, our study does not support significant causal associations of genetically determined diet-derived circulating antioxidants of vitamins E and C, retinol, β-carotene, and lycopene, with the risk of six major mental disorders. Therefore, for healthy adults without nutritional deficiency and pathological damage, simply taking antioxidants to increase blood antioxidant levels may not have a significant protective effect on the prevention of major mental disorders. In the future, large-scale GWASs are needed to further validate our current findings by utilizing additional genetic variants and more samples.

## Figures and Tables

**Table 1:** The summary of instrumental variables for diet-derived absolute circulating antioxidants and antioxidant metabolites.

Trait	Sample Size	*p*-Value	LD	No. of SNPs	Explained Variance (%)	Unit	PMID
Absolute circulating antioxidants							
Ascorbate	52,018	5 × 10^−8^	0.001	10	1.7	µmol/L	33203707
Retinol	5006	5 × 10^−8^	0.001	2	2.3	µg/L in ln-transformed scale	21878437
β-Carotene	2344	5 × 10^−8^	0.001	2	4.8	µg/L in ln-transformed scale	23134893
Lycopene	441	5 × 10^−6^	0.001	5	30.1	µg/dL	26861389
Circulating antioxidant metabolites							
α-Tocopherol	7725	1 × 10^−5^	0.001	11	6.8	log10-transformed metabolites concentration	24816252
γ-Tocopherol	6226	1 × 10^−5^	0.001	13	9.8	log10-transformed metabolites concentration	24816252
Ascorbate	2085	1 × 10^−5^	0.001	14	21.7	log10-transformed metabolites concentration	24816252
Retinol	1960	1 × 10^−5^	0.001	26	20.6	log10-transformed metabolites concentration	28263315

Abbreviations: LD, linkage disequilibrium.

## Data Availability

All relevant data are within the manuscript and its supporting information files. The summary-level data for diet-derived circulating antioxidants are available in the corresponding original studies. The summary-level data for six major mental disorders are available at the Psychiatric Genomics Consortium (PGC) website (https://www.med.unc.edu/pgc/results-and-downloads/ (accessed on 25 June 2022)), the Pan-UKB project (https://pan.ukbb.broadinstitute.org/ (accessed on 25 June 2022)), and the FinnGen study (https://FinnGengen.gitbook.io/documentation/ (accessed on 25 June 2022)).

## Supplementary Materials

The following supporting information can be downloaded at: https://www.mdpi.com/article/10.3390/antiox12010162/s1, Table S1. The summary information of the studies used for instrumental variables extraction of absolute circulating antioxidants. Table S2. The summary statistics of the six major mental disorders. Table S3. Minimum detectable OR with sufficient power in MR analysis. Table S4. The causal effect estimates of the associations between instrumental variables for absolute blood antioxidants and the risk of six major psychiatric disorders. Table S5. The causal effect estimates of the associations between instrumental variables for antioxidant metabolite concentrations and the risk of six major psychiatric disorders. Table S6. The MR analysis results of the causal effects of absolute ascorbate levels on major mental disorders. Table S7. The MR analysis results of the causal effects of absolute retinol levels on major mental disorders. Table S8. The MR analysis results of the causal effects of absolute β-carotene levels on major mental disorders. Table S9. The MR analysis results of the causal effects of absolute lycopene levels on major mental disorders. Table S10. The MR analysis results of the causal effects of α-tocopherol metabolites on major mental disorders. Table S11. The MR analysis results of the causal effects of γ-tocopherol metabolites on major mental disorders. Table S12. The MR analysis results of the causal effects of ascorbate metabolites on major mental disorders. Table S13. The MR analysis results of the causal effects of retinol metabolites on major mental disorders. Figure S1. The MR analyses for absolute circulating antioxidant levels on the risk of major mental disorders after removing outliers. Figure S2. The causal effects of four absolute circulating antioxidant levels on the risk of depression. Figure S3. The MR analyses for circulating antioxidant metabolites on the risk of major mental disorders after removing outliers. Figure S4. The causal effects of four circulating antioxidant metabolites on the risk of depression. Reference [63] is cited in supplementary materials.
