# Supplementary material for: Dissecting Causal Associations of Diet-Derived Circulating Antioxidants with Six Major Mental Disorders: A Mendelian Randomization Study
Source: Antioxidants (Basel). 2023 Jan 10;12(1):162. doi: 10.3390/antiox12010162 (PMC9855039; doi:10.3390/antiox12010162)
Supplement: Supplementary file 1 [file antioxidants-12-00162-s001.zip › Supplemental Figures S1-S4.pdf]

Supplementary Figures

Absolute circulating antioxidants	Outcome	No. SNPs	Odds ratio (95%CI)	*P*-value	Weight
Ascorbate	**BIP**				
	PGC†	9	1.025 (0.927–1.133)	0.633	80.4%
	UK Biobank	10	0.872 (0.577–1.318)	0.515	4.7%
	FinnGen†	9	0.962 (0.762–1.215)	0.747	14.8%
	**Overall effect (Fixed Model)**		**1.008 (0.921–1.102)**	**0.871**	**100.0%**
	Heterogeneity: I^2 = 0.0%, *P* = 0.694				
Ascorbate	**SCZ**				
	PGC†	9	0.901 (0.788–1.030)	0.438	82.7%
	UK Biobank	10	1.138 (0.522–2.478)	0.438	2.4%
	FinnGen	10	0.898 (0.655–1.230)	0.989	14.9%
	**Overall effect (Fixed Model)**		**0.906 (0.802–1.023)**	**0.111**	**100.0%**
	Heterogeneity: I^2 = 0.0%, *P* = 0.844				
Ascorbate	**Depression**				
	PGC & UK Biobank	10	1.030 (0.966–1.097)	0.368	69.7%
	FinnGen†	9	1.051 (0.955–1.158)	0.309	30.3%
	**Overall effect (Fixed Model)**		**1.036 (0.983–1.093)**	**0.190**	**100.0%**
	Heterogeneity: I^2 = 0.0%, *P* = 0.724				

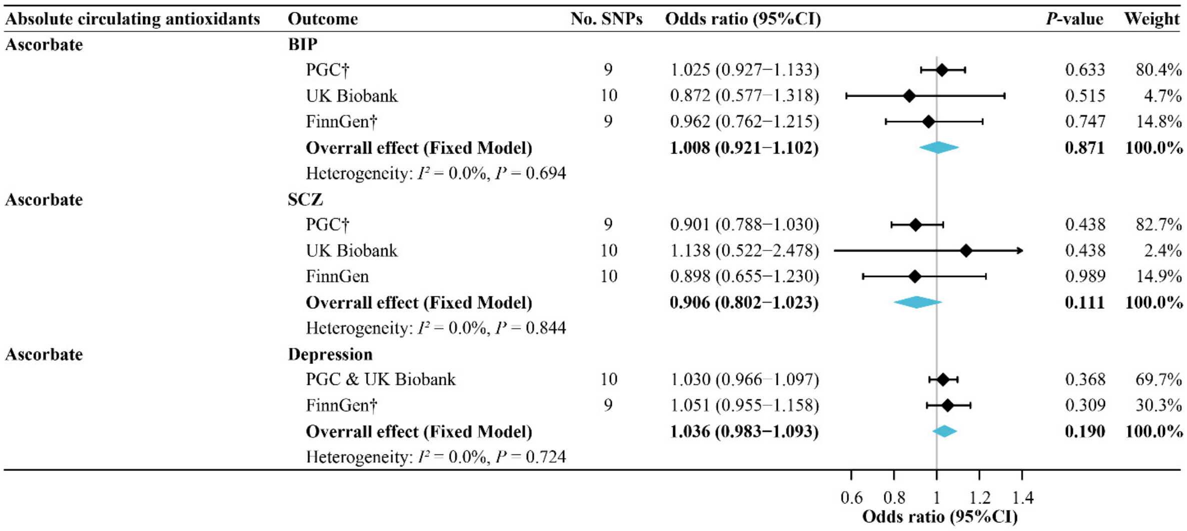

Figure S1. The MR analyses for absolute circulating antioxidant levels on the risk of major mental disorders after removing outliers. The ORs are scaled per µmol/L increase in ascorbate. †: MR analysis results after removing 1 outlier by MR-PRESSO.

Absolute circulating antioxidants	No. SNPs	Odds ratio (95%CI)	*P*-value	Weight
**Ascorbate**				
PGC & UK Biobank	10	1.03 (0.966–1.097)	0.368	83.2%
FinnGen	10	1.09 (0.946–1.257)	0.232	16.8%
**Overall effect (Fixed Model)**		**1.04 (0.981–1.102)**	**0.190**	**100.0%**
Heterogeneity: I^2 = 0.0%, *P* = 0.471				
**Retinol**				
PGC & UK Biobank	2	1.008 (0.956–1.063)	0.773	38.1%
FinnGen	2	1.038 (0.996–1.083)	0.079	61.9%
**Overall effect (Fixed Model)**		**1.027 (0.993–1.061)**	**0.119**	**100.0%**
Heterogeneity: I^2 = 0.0%, *P* = 0.390				
**β-Carotene**				
PGC & UK Biobank	2	1.004 (0.952–1.059)	0.879	80.0%
FinnGen	2	0.911 (0.820–1.013)	0.084	20.0%
**Overall effect (Fixed Model)**		**0.985 (0.939–1.032)**	**0.524**	**100.0%**
Heterogeneity: I^2 = 61.7%, *P* = 0.106				
**Lycopene**				
PGC & UK Biobank	5	1.004 (0.986–1.022)	0.693	81.4%
FinnGen	5	0.973 (0.938–1.011)	0.158	18.6%
**Overall effect (Fixed Model)**		**0.998 (0.982–1.014)**	**0.800**	**100.0%**
Heterogeneity: I^2 = 52.0%, *P* = 0.149				

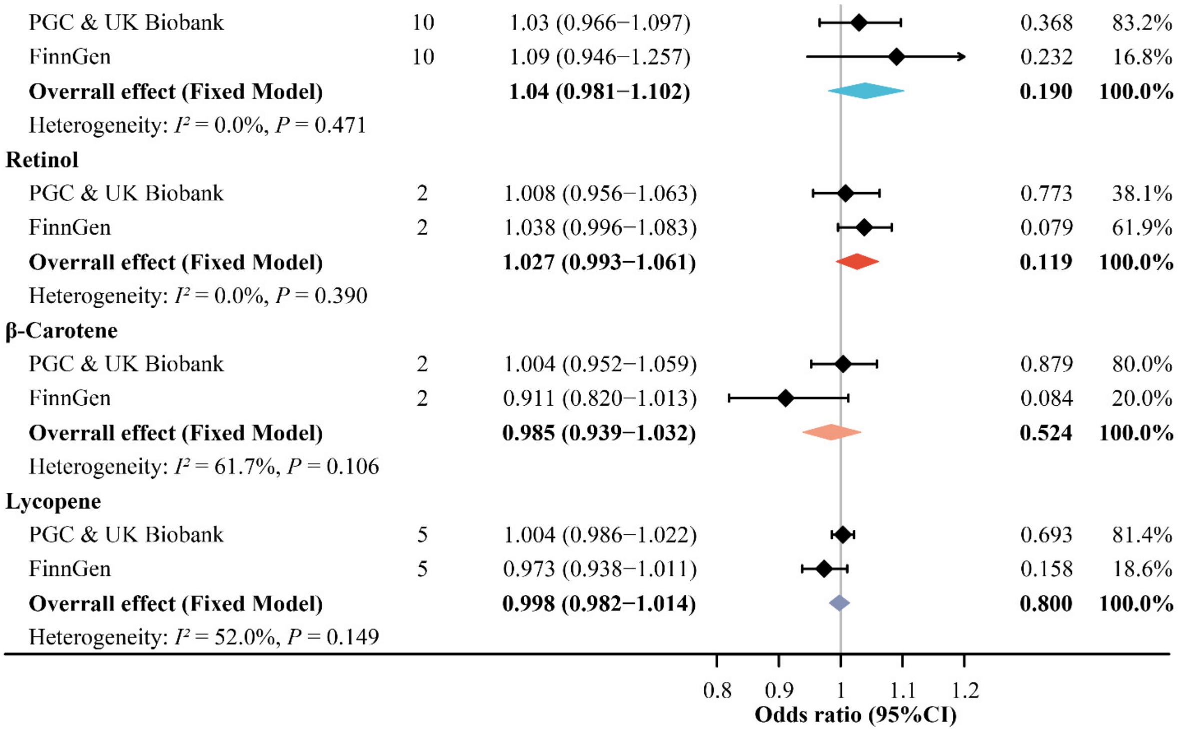

Figure S2. The causal effects of four absolute circulating antioxidant levels on the risk of depression. The ORs are scaled per µmol/L increase in ascorbate, per 0.1 unit increase in ln-transformed retinol, per unit increase in ln-transformed β-carotene, and per µg/dL increase in lycopene.

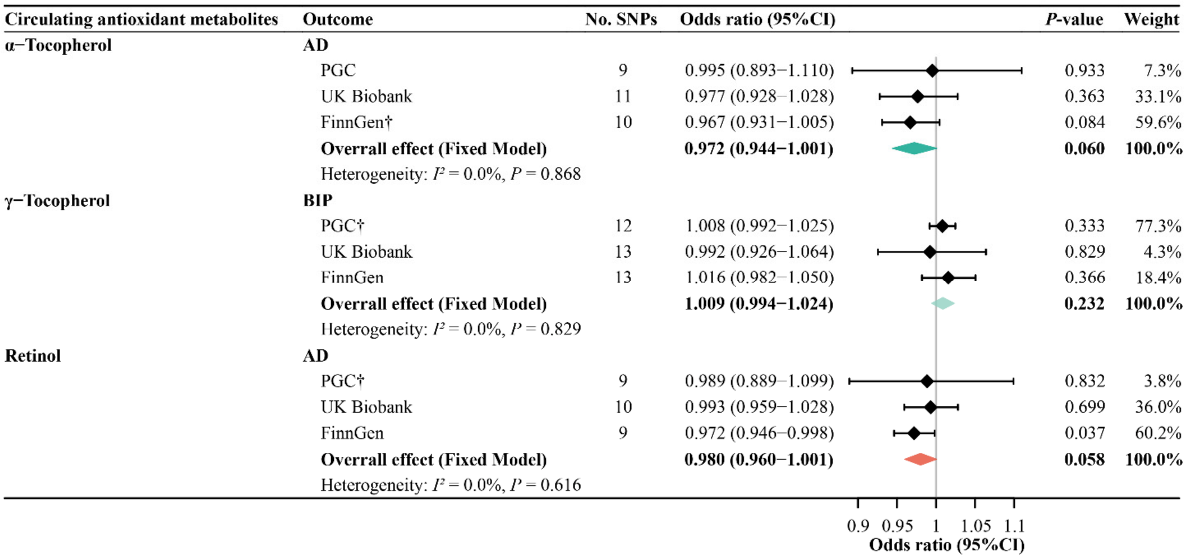

Figure S3. The MR analyses for circulating antioxidant metabolites on the risk of major mental disorders after removing outliers. The ORs are scaled per 0.1 unit increase in log-transformed α-tocopherol and γ-tocopherol, and per unit increase in log-transformed retinol. †: MR analysis results after removing 1 outlier by MR-PRESSO.

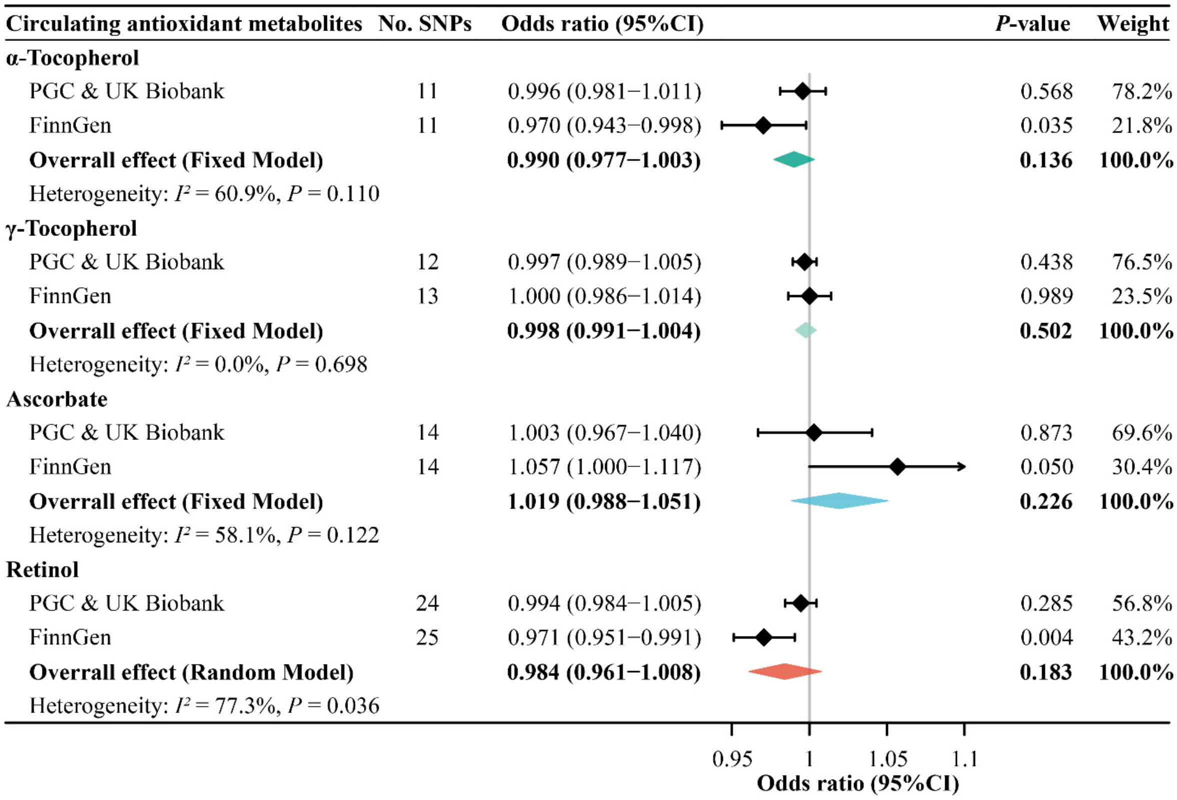

Figure S4. The causal effects of four circulating antioxidant metabolites on the risk of depression. The ORs are scaled per 0.1 unit increase in log-transformed α-tocopherol and γ-tocopherol, and per unit increase in log-transformed ascorbate and retinol
