# Supplementary material for: Dissecting Causal Associations of Diet-Derived Circulating Antioxidants with Six Major Mental Disorders: A Mendelian Randomization Study
Source: Antioxidants (Basel). 2023 Jan 10;12(1):162. doi: 10.3390/antiox12010162 (PMC9855039; doi:10.3390/antiox12010162)
Supplement: Supplementary file 1 [file antioxidants-12-00162-s001.zip › Supplemental Tables S1-S13.pdf]

Supplementary Tables

Index

Table S1. The summary information of the studies used for instrumental variables extraction of absolute circulating antioxidants....... 1
Table S2. The summary statistics of the six major mental disorders....... 2
Table S3. Minimum detectable OR with sufficient power in MR analysis....... 3
Table S4. The causal effect estimates of the associations between instrumental variables for absolute blood antioxidants and the risk of six major psychiatric disorders....... 4
Table S5. The causal effect estimates of the associations between instrumental variables for antioxidant metabolite concentrations and the risk of six major psychiatric disorders....... 7
Table S6. The MR analysis results of the causal effects of absolute ascorbate levels on major mental disorders....... 14
Table S7. The MR analysis results of the causal effects of absolute retinol levels on major mental disorders....... 17
Table S8. The MR analysis results of the causal effects of absolute β-carotene levels on major mental disorders....... 19
Table S9. The MR analysis results of the causal effects of absolute lycopene levels on major mental disorders....... 21
Table S10. The MR analysis results of the causal effects of α-tocopherol metabolites on major mental disorders....... 24
Table S11. The MR analysis results of the causal effects of γ-tocopherol metabolites on major mental disorders....... 27
Table S12. The MR analysis results of the causal effects of ascorbate metabolites on major mental disorders....... 30
Table S13. The MR analysis results of the causal effects of retinol metabolites on major mental disorders....... 33

Table S1. The summary information of the studies used for instrumental variables extraction of absolute circulating antioxidants.

**Absolute circulating antioxidants**	**Sample size**	**Age**	**Sex (male)**	**Antioxidant concentration**
**α-Tocopherol (mg/L)** [1]				
ATBC	4,014	58.1±5.0	100%	11.9 ±3.4
**Ascorbate (µmol/L)** [2]				
Fenland GWAS	1,349	45±7	44%	66.2±21.3
Fenland UKBB	8,391	49±7	47%	68.6±21.5
Fenland Core Exome	1,031	51±7	45%	68.3±21.8
InterAct subcohort GWAS	3,521	51±9	35%	42.8±19
InterAct subcohort core-exome	6,504	53±9	38%	42.9±19.1
InterAct non-subcohort GWAS	2,944	55±8	48%	36.4±17.6
InterAct non-subcohort core-exome	3,872	56±7	52%	36.5±18.9
EPIC-Norfolk GWAS	16,756	59±9	47%	53.8±20.2
EPIC-CVD subcohort	885	53±12	41%	41.0±21.0
EPIC-CVD non-subcohort	6,765	57±8	55%	37.9±20.8
**Retinol (µg/dL)** [3]				
ATBC	4,014	58.1±5.0	100%	572 (796-654)
PLCO	992	64.6 ± 4.9	100%	672 (562-794)
**β-Carotene (µg/L)** [4]				
NHS	2,344	58.8±6.4	0%	303±258
**Lycopene (µg/dL)** [5]				
HAPI	441	43.1±13.0	58%	39.2±19.9

ATBC, Beta-Carotene Cancer Prevention Study; EPIC-Norfolk, the Norfolk arm of the European Prospective Investigation on Cancer study; EPIC, European Prospective Investigation into Cancer and Nutrition; PLCO, Prostate, Lung, Colorectal, and Ovarian Cancer Screening Trial; NHS, the Nurses' Health Study; HAPI, Heredity and Phenotype Intervention Heart Study.

Table S2. The summary statistics of the six major mental disorders.

Trait	GWAS data source	Sample Size	Cases	Controls	Ancestry	Publication
**Primary outcome**						
AD	ANGST Consortium	17,310	33%	67%	All European	Otowa et al, 2016[6]
AD	UK Biobank-Phecode (300.1)	379,635	9,705	369,930	All European	Pan-UKB, 2018
AD	FinnGen-F5_ALLANXIOUS	297,176	18,358	278,818	All European	Kurki et al, 2022[7]
MDD	PGC	173,005	59,851	113,154	All European	Wray et al, 2018[8]
MDD	UK Biobank-Phecode (296.22)	370,408	478	369,930	All European	Pan-UKB, 2018
BIP	PGC	413,466	41,917	371,549	All European	Mullins et al, 2021[9]
BIP	UK Biobank-Phecode (296.1)	371,187	1,257	369,930	All European	Pan-UKB, 2018
BIP	FinnGen-F5_BIPO	277,143	5,763	271,380	All European	Kurki et al, 2022[7]
SCZ	PGC	127,906	52,017	75,889	All European	Trubetskoy et al, 2022[10]
SCZ	UK Biobank-Phecode (295.1)	370,631	701	369,930	All European	Pan-UKB, 2018
SCZ	FinnGen-F5_SCHZPHR	303,546	6,050	297,496	All European	Kurki et al, 2022[7]
PTSD	PGC	174,659	23,212	151,447	≥90% European	Nievergelt et al, 2019[11]
PTSD	UK Biobank-Phecode (300.9)	370,117	187	369,930	All European	Pan-UKB, 2018
PTSD	FinnGen-F5_PTSD	280,457	1,639	278,818	All European	Kurki et al, 2022[7]
OCD	IOCDF-GC & OCGAS	9,725	2,688	7,037	>85% European	IOCDF-GC and OCGAS, 2017[12]
OCD	UK Biobank-Phecode (300.3)	370,123	193	369,930	All European	Pan-UKB, 2018
OCD	FinnGen-F5_OCD	280,284	1,466	278,818	All European	Kurki et al, 2022[7]
**Secondary outcome**						
Depression	PGC & UK Biobank	500,199	170,756	329,443	All European	Howard et al, 2019[13]
Depression	FinnGen-F5_DEPRESSIO	305,192	33,812	271,380	All European	Kurki et al, 2022[7]

AD, anxiety disorders; MDD, major depressive disorder; BIP, bipolar disorder; SCZ, schizophrenia; PTSD, post-traumatic stress disorder; OCD, obsessive-compulsive disorder; PGC, Psychiatric Genomics Consortium; ANGST, Anxiety NeuroGenetics Study; IOCDF-GC, International Obsessive-Compulsive Disorder Foundation Genetics Collaborative; OCGAS, OCD Collaborative Genetics Association Study.

Pan-UKB project: https://pan.ukbb.broadinstitute.org/

FinnGen study: https://FinnGengen.gitbook.io/documentation/

Table S3. Minimum detectable OR with sufficient power in MR analysis.

Outcome	Absolute circulating antioxidants*				Circulating antioxidant metabolites*			
	Ascorbate	Retinol	β-Carotene	Lycopene	α-Tocopherol	γ-Tocopherol	Ascorbate	Retinol
**Primary**								
**AD**								
ANGST Consortium	0.72 / 1.38	0.75 / 1.33	0.82 / 1.22	0.92 / 1.09	0.84 / 1.18	0.87 / 1.15	0.91 / 1.1	0.91 / 1.1
UK Biobank	0.82 / 1.22	0.84 / 1.19	0.88 / 1.13	0.95 / 1.05	0.99 / 1.01	0.92 / 1.09	0.94 / 1.06	0.94 / 1.06
FinnGen	0.86 / 1.17	0.88 / 1.14	0.91 / 1.1	0.96 / 1.04	0.99 / 1.01	0.94 / 1.07	0.96 / 1.05	0.96 / 1.05
**MDD**								
PGC	0.9 / 1.11	0.91 / 1.1	0.94 / 1.07	0.97 / 1.03	0.99 / 1.01	0.96 / 1.05	0.97 / 1.03	0.97 / 1.03
UK Biobank	0.5 / 1.99	0.54 / 1.85	0.63 / 1.59	0.81 / 1.23	0.96 / 1.04	0.71 / 1.41	0.78 / 1.28	0.78 / 1.28
**BIP**								
PGC	0.9 / 1.11	0.91 / 1.1	0.94 / 1.07	0.97 / 1.03	0.99 / 1.01	0.96 / 1.05	0.97 / 1.03	0.97 / 1.03
UK Biobank	0.62 / 1.61	0.66 / 1.52	0.74 / 1.36	0.87 / 1.14	0.97 / 1.03	0.8 / 1.25	0.85 / 1.17	0.85 / 1.17
FinnGen	0.78 / 1.29	0.8 / 1.25	0.85 / 1.17	0.94 / 1.07	0.99 / 1.01	0.89 / 1.12	0.93 / 1.08	0.92 / 1.08
**SCZ**								
PGC	0.89 / 1.13	0.9 / 1.11	0.93 / 1.08	0.97 / 1.03	0.99 / 1.01	0.95 / 1.05	0.97 / 1.04	0.97 / 1.04
UK Biobank	0.55 / 1.81	0.59 / 1.7	0.67 / 1.48	0.84 / 1.19	0.97 / 1.03	0.75 / 1.34	0.81 / 1.23	0.81 / 1.23
FinnGen	0.78 / 1.28	0.81 / 1.24	0.86 / 1.17	0.94 / 1.07	0.99 / 1.01	0.9 / 1.12	0.93 / 1.08	0.93 / 1.08
**PTSD**								
PGC	0.87 / 1.15	0.88 / 1.13	0.92 / 1.09	0.97 / 1.04	0.99 / 1.01	0.94 / 1.06	0.96 / 1.04	0.96 / 1.04
UK Biobank	0.39 / 2.57	0.43 / 2.35	0.52 / 1.94	0.73 / 1.37	0.94 / 1.06	0.6 / 1.66	0.69 / 1.44	0.69 / 1.45
FinnGen	0.65 / 1.53	0.69 / 1.46	0.76 / 1.32	0.89 / 1.13	0.98 / 1.02	0.82 / 1.22	0.87 / 1.15	0.87 / 1.15
**OCD**								
IOCDF-GC & OCGAS	0.65 / 1.55	0.68 / 1.46	0.76 / 1.31	0.89 / 1.12	0.98 / 1.02	0.82 / 1.21	0.88 / 1.14	0.87 / 1.15
UK Biobank	0.39 / 2.54	0.43 / 2.33	0.73 / 1.37	0.52 / 1.92	0.94 / 1.06	0.61 / 1.64	0.7 / 1.43	0.69 / 1.44
FinnGen	0.64 / 1.56	0.67 / 1.48	0.75 / 1.33	0.88 / 1.13	0.98 / 1.02	0.81 / 1.23	0.86 / 1.16	0.86 / 1.16
**Secondary**								
**Depression**								
PGC & UK Biobank	0.94 / 1.07	0.95 / 1.06	0.96 / 1.04	0.98 / 1.02	0.99 / 1.01	0.97 / 1.03	0.98 / 1.02	0.98 / 1.02
UK Biobank	0.89 / 1.13	0.9 / 1.11	0.93 / 1.07	0.97 / 1.03	0.99 / 1.01	0.95 / 1.05	0.97 / 1.04	0.97 / 1.04

AD, anxiety disorders; MDD, major depressive disorder; BIP, bipolar disorder; SCZ, schizophrenia; PTSD, post-traumatic stress disorder; OCD, obsessive-compulsive disorder; PGC, Psychiatric Genomics Consortium; ANGST, Anxiety NeuroGenetics Study; IOCDF-GC, International Obsessive-Compulsive Disorder Foundation Genetics Collaborative; OCGAS, OCD Collaborative Genetics Association Study.

*: The data in the table were the minimum detectable OR (protective effect / risk effect).

Table S4. The causal effect estimates of the associations between instrumental variables for absolute blood antioxidants and the risk of six major psychiatric disorders.

Trait	SNP	Gene	Effect allele	F	Exposure		Primary outcome												Depression	
							AD		MDD		BIP		SCZ		PTSD		OCD			
					Beta	SE	Beta	SE	Beta	SE	Beta	SE	Beta	SE	Beta	SE	Beta	SE	Beta	SE
**The largest GWAS published to date***																				
ascorbate	rs10051765	RGS14	C	31	0.039	0.007	-0.002	0.031	-0.001	0.009	0.000	0.010	-0.014	0.009	0.001	0.016	-0.003	0.037	-0.001	0.005
ascorbate	rs10136000	AKT1	A	33	0.040	0.007	-0.061	0.044	0.003	0.010	0.020	0.011	0.017	0.010	-0.009	0.018	0.054	0.045	0.001	0.005
ascorbate	rs117885456	SNRPF	A	42	0.078	0.012	0.020	0.075	-0.022	0.016	0.010	0.017	-0.007	0.016	0.012	0.030	0.054	0.077	-0.010	0.008
ascorbate	rs13028225	SLC23A3	T	128	0.102	0.009	0.015	0.040	-0.010	0.012	0.013	0.014	-0.031	0.013	-0.003	0.021	0.070	0.050	-0.005	0.006
ascorbate	rs174547	FADS1	C	26	0.036	0.007	-0.008	0.030	0.020	0.008	0.068	0.010	0.024	0.009	-0.003	0.016	0.015	0.036	0.015	0.005
ascorbate	rs2559850	CHPT1	A	93	0.058	0.006	-0.014	0.028	0.016	0.008	0.004	0.010	0.015	0.009	0.014	0.015	-0.027	0.035	0.002	0.004
ascorbate	rs33972313	SLC23A1	C	400	0.360	0.018	-0.097	0.105	-0.015	0.022	-0.029	0.027	-0.035	0.025	-0.022	0.042	0.031	0.103	0.004	0.012
ascorbate	rs56738967	MAF	C	34	0.041	0.007	0.010	0.028	0.001	0.008	0.000	0.010	-0.011	0.009	0.020	0.016	-0.070	0.036	0.007	0.005
ascorbate	rs6693447	RER1	T	42	0.039	0.006	-0.014	0.030	0.015	0.009	0.007	0.010	-0.016	0.009	0.011	0.015	0.012	0.034	0.002	0.005
ascorbate	rs9895661	BCAS3	T	62	0.063	0.008	-0.073	0.042	0.004	0.011	0.005	0.012	-0.004	0.011	0.010	0.019	0.013	0.044	0.012	0.006
retinol	rs10882272	RBP4	C	56	-0.030	0.004	0.051	0.031	0.002	0.008	-0.004	0.010	-0.012	0.009	-0.023	0.015	-0.010	0.034	0.006	0.004
retinol	rs1667255	TTR	C	56	0.030	0.004	-0.009	0.027	0.017	0.008	-0.008	0.010	0.012	0.008	0.008	0.015	0.040	0.034	0.011	0.004
carotene	rs6564851	BCMO1	G	99	0.149	0.015	0.049	0.027	0.006	0.008	-0.008	0.009	0.003	0.009	-0.010	0.015	-0.042	0.034	0.003	0.004
carotene	rs7501331	BCMO1	T	18	-0.067	0.016	-0.003	0.034	0.014	0.009	0.003	0.011	0.000	0.010	0.013	0.017	-0.032	0.041	0.008	0.005
lycopene	rs2232315	G6PC2	A	24	0.740	0.150	-	-	-0.039	0.036	-0.041	0.043	-0.026	0.036	-0.026	0.070	-0.235	0.230	-0.016	0.017
lycopene	rs341075	-	A	26	-0.870	0.170	0.053	0.060	0.006	0.025	-0.039	0.028	0.020	0.026	-0.006	0.047	-0.131	0.103	0.007	0.013
lycopene	rs4635297	BC039545	A	27	0.260	0.050	0.029	0.042	0.036	0.011	-0.011	0.013	0.024	0.012	0.007	0.021	0.044	0.044	0.010	0.006
lycopene	rs6108801	-	C	28	-0.480	0.090	0.046	0.039	-0.011	0.022	-0.008	0.027	0.011	0.025	0.011	0.041	0.043	0.097	-0.006	0.012
lycopene	rs7680948	SETD7	A	40	-0.190	0.030	-0.050	0.033	-0.015	0.009	0.003	0.011	-0.006	0.010	-0.002	0.017	0.034	0.040	-0.003	0.005
**UK Biobank**																				
ascorbate	rs10051765	RGS14	C	31	0.039	0.007	-0.009	0.016	0.198	0.069	-0.020	0.043	0.039	0.057	0.108	0.110	0.083	0.108	-	-
ascorbate	rs10136000	AKT1	A	33	0.040	0.007	-0.018	0.016	0.021	0.072	-0.065	0.045	-0.001	0.060	-0.126	0.115	0.059	0.114	-	-

ascorbate	rs117885456	SNRPF	A	42	0.078	0.012	-0.003	0.026	-0.034	0.115	0.033	0.072	0.261	0.095	0.192	0.183	-0.235	0.181	-	-
ascorbate	rs13028225	SLC23A3	T	128	0.102	0.009	-0.013	0.021	-0.044	0.093	-0.035	0.058	0.061	0.077	0.199	0.149	-0.162	0.147	-	-
ascorbate	rs174547	FADS1	C	26	0.036	0.007	0.005	0.015	0.018	0.068	0.049	0.042	0.052	0.056	0.126	0.109	-0.029	0.107	-	-
ascorbate	rs2559850	CHPT1	A	93	0.058	0.006	-0.001	0.015	-0.059	0.067	0.016	0.042	-0.145	0.055	0.042	0.106	-0.026	0.105	-	-
ascorbate	rs33972313	SLC23A1	C	400	0.360	0.018	-0.035	0.040	0.357	0.176	-0.164	0.110	-0.080	0.146	0.087	0.281	0.349	0.276	-	-
ascorbate	rs56738967	MAF	C	34	0.041	0.007	-0.006	0.016	0.068	0.070	0.072	0.043	0.001	0.058	-0.052	0.111	-0.163	0.110	-	-
ascorbate	rs6693447	RER1	T	42	0.039	0.006	-0.002	0.015	0.193	0.065	0.010	0.041	0.038	0.054	0.082	0.104	-0.072	0.102	-	-
ascorbate	rs9895661	BCAS3	T	62	0.063	0.008	0.025	0.020	0.024	0.086	0.036	0.054	0.076	0.072	0.039	0.138	0.153	0.136	-	-
retinol	rs10882272	RBP4	C	56	-0.030	0.004	0.019	0.015	0.078	0.067	0.085	0.042	-0.027	0.055	-0.121	0.106	-0.036	0.105	-	-
retinol	rs1667255	TTR	C	56	0.030	0.004	0.013	0.015	-0.048	0.067	0.006	0.042	-0.041	0.055	0.090	0.106	-0.107	0.105	-	-
carotene	rs6564851	BCMO1	G	99	0.149	0.015	0.024	0.015	0.057	0.065	-0.055	0.040	-0.026	0.054	0.263	0.104	0.018	0.102	-	-
carotene	rs7501331	BCMO1	T	18	-0.067	0.016	0.007	0.017	-0.005	0.076	-0.007	0.047	0.076	0.063	0.016	0.121	-0.164	0.119	-	-
lycopene	rs2232315	G6PC2	A	24	0.740	0.150	0.044	0.054	0.051	0.238	-0.319	0.148	-0.359	0.198	-0.152	0.379	0.247	0.375	-	-
lycopene	rs341075	-	A	26	-0.870	0.170	-0.040	0.044	-0.434	0.191	-0.061	0.120	-0.086	0.159	-0.383	0.306	0.390	0.302	-	-
lycopene	rs4635297	BC039545	A	27	0.260	0.050	0.012	0.019	-0.005	0.083	-0.005	0.052	0.003	0.068	0.027	0.132	0.108	0.130	-	-
lycopene	rs6108801	-	C	28	-0.480	0.090	-0.011	0.040	-0.048	0.174	0.013	0.109	-0.120	0.144	0.030	0.278	0.453	0.275	-	-
lycopene	rs7680948	SETD7	A	40	-0.190	0.030	0.004	0.017	-0.018	0.073	0.036	0.045	0.011	0.060	0.053	0.116	0.098	0.115	-	-
**FinnGen study**																				
ascorbate	rs10051765	RGS14	C	31	0.039	0.007	0.001	0.011	-	-	0.037	0.021	0.026	0.029	-0.068	0.036	-0.007	0.038	0.009	0.009
ascorbate	rs10136000	AKT1	A	33	0.040	0.007	0.007	0.012	-	-	-0.015	0.023	-0.002	0.031	0.033	0.039	0.019	0.041	-0.004	0.009
ascorbate	rs117885456	SNRPF	A	42	0.078	0.012	-0.002	0.020	-	-	0.044	0.038	0.062	0.052	-0.075	0.065	-0.061	0.069	-0.007	0.015
ascorbate	rs13028225	SLC23A3	T	128	0.102	0.009	-0.005	0.016	-	-	0.032	0.028	-0.033	0.039	-0.066	0.049	0.027	0.052	0.008	0.012
ascorbate	rs174547	FADS1	C	26	0.036	0.007	0.023	0.011	-	-	0.090	0.021	-0.019	0.028	0.032	0.036	0.075	0.038	0.034	0.009
ascorbate	rs2559850	CHPT1	A	93	0.058	0.006	0.023	0.011	-	-	0.010	0.021	0.002	0.029	0.002	0.036	0.044	0.038	0.015	0.009
ascorbate	rs33972313	SLC23A1	C	400	0.360	0.018	0.027	0.038	-	-	-0.135	0.070	-0.049	0.098	0.214	0.120	0.065	0.127	0.012	0.029
ascorbate	rs56738967	MAF	C	34	0.041	0.007	0.012	0.012	-	-	-0.018	0.022	-0.039	0.030	0.018	0.038	0.016	0.041	-0.009	0.009
ascorbate	rs6693447	RER1	T	42	0.039	0.006	-0.011	0.011	-	-	0.009	0.021	0.021	0.028	-0.053	0.035	-0.065	0.038	0.010	0.009
ascorbate	rs9895661	BCAS3	T	62	0.063	0.008	-0.003	0.014	-	-	-0.023	0.026	-0.032	0.036	-0.025	0.045	0.039	0.048	-0.006	0.011
retinol	rs10882272	RBP4	C	56	-0.030	0.004	0.031	0.012	-	-	-0.013	0.021	0.060	0.029	0.056	0.037	0.013	0.039	-0.001	0.009

retinol	rs1667255	TTR	C	56	0.030	0.004	0.021	0.012	-	-	0.007	0.023	0.048	0.031	0.005	0.039	-0.012	0.042	0.023	0.009
carotene	rs6564851	BCMO1	G	99	0.149	0.015	0.000	0.012	-	-	-0.016	0.021	0.028	0.029	0.042	0.036	-0.035	0.039	-0.011	0.009
carotene	rs7501331	BCMO1	T	18	-0.067	0.016	0.000	0.012	-	-	-0.006	0.022	-0.032	0.031	-0.025	0.038	-0.061	0.041	0.012	0.009
lycopene	rs2232315	G6PC2	A	24	0.740	0.150	-0.156	0.079	-	-	-0.052	0.142	0.089	0.195	0.153	0.249	-0.296	0.260	-0.067	0.060
lycopene	rs341075	-	A	26	-0.870	0.170	0.040	0.030	-	-	0.014	0.055	-0.053	0.076	0.004	0.095	-0.005	0.100	0.000	0.022
lycopene	rs4635297	BC039545	A	27	0.260	0.050	-0.021	0.015	-	-	-0.002	0.027	0.086	0.037	0.028	0.046	-0.025	0.049	-0.002	0.011
lycopene	rs6108801	-	C	28	-0.480	0.090	0.042	0.050	-	-	-0.059	0.092	-0.047	0.128	-0.183	0.160	0.018	0.171	0.047	0.038
lycopene	rs7680948	SETD7	A	40	-0.190	0.030	0.005	0.013	-	-	0.026	0.024	0.059	0.033	0.038	0.042	0.050	0.044	0.021	0.010

AD, anxiety disorders; MDD, major depressive disorder; BIP, bipolar disorder; SCZ, schizophrenia; PTSD, post-traumatic stress disorder; OCD, obsessive-compulsive disorder;

*: The largest GWAS study to date for AD from ANGST Consortium, for MDD, BIP, SCZ and PTSD from PGC, and for OCD from IOCDF-GC & OCGAS. The following table S5 description is the same as this table description

Table S5. The causal effect estimates of the associations between instrumental variables for antioxidant metabolite concentrations and the risk of six major psychiatric disorders.

Trait	SNP	Gene	Effect allele	F	Exposure		Primary outcome												Depression	
							AD		MDD		BIP		SCZ		PTSD		OCD			
					Beta	SE	Beta	SE	Beta	SE	Beta	SE	Beta	SE	Beta	SE	Beta	SE	Beta	SE
**The largest GWAS published to date***																				
α-tocopherol	rs10163969	-	T	19	-0.035	0.008	0.043	0.142	0.044	0.022	0.016	0.022	0.003	0.020	0.062	0.037	0.096	0.080	0.003	0.011
α-tocopherol	rs10245705	LOC105375199	T	27	-0.066	0.013	-	-	0.013	0.032	-0.010	0.037	0.064	0.033	-0.031	0.057	-0.224	0.140	0.004	0.016
α-tocopherol	rs10935814	-	A	20	-0.037	0.008	-0.015	0.045	-0.006	0.012	-0.007	0.014	0.011	0.013	-0.009	0.023	-0.003	0.052	-0.004	0.007
α-tocopherol	rs11145330	-	C	23	-0.032	0.007	-0.028	0.041	-0.003	0.012	0.015	0.015	0.006	0.014	-0.042	0.023	0.040	0.054	0.000	0.007
α-tocopherol	rs11992435	-	G	21	-0.033	0.007	-0.002	0.067	-0.006	0.017	-0.036	0.020	-0.001	0.018	0.032	0.032	-0.073	0.071	-0.005	0.009
α-tocopherol	rs1404410	-	G	21	0.024	0.005	0.009	0.032	-0.002	0.010	0.014	0.011	0.025	0.011	0.017	0.018	0.016	0.041	0.000	0.005
α-tocopherol	rs1532701	SLC6A2	A	21	0.014	0.003	-0.025	0.026	0.007	0.008	-0.004	0.009	-0.011	0.009	0.005	0.015	-0.011	0.034	0.000	0.004
α-tocopherol	rs2074731	SF3A1	A	22	-0.018	0.004	0.043	0.035	0.011	0.011	0.004	0.012	-0.001	0.011	-0.014	0.020	0.058	0.044	0.010	0.006
α-tocopherol	rs261342	LIPC/LIPC-AS1	C	21	-0.017	0.004	-0.023	0.036	-0.005	0.010	-0.013	0.011	-0.016	0.010	0.000	0.018	-0.005	0.041	0.004	0.005
α-tocopherol	rs7238006	-	C	24	-0.028	0.006	0.021	0.050	-0.020	0.015	-0.008	0.017	-0.004	0.016	0.012	0.027	0.128	0.062	0.000	0.008
α-tocopherol	rs7930821	ZDHHC13	T	20	0.067	0.015	-	-	-0.019	0.026	0.010	0.031	-0.033	0.029	0.025	0.051	-0.242	0.119	-0.011	0.015
γ-tocopherol	rs10077932	-	T	21	-0.040	0.009	0.036	0.041	0.000	0.011	-0.033	0.012	-0.003	0.011	-0.031	0.020	0.029	0.048	-0.012	0.006
γ-tocopherol	rs1013104	-	T	21	-0.021	0.005	-0.053	0.026	-0.007	0.008	0.003	0.009	0.000	0.009	-0.033	0.015	-0.030	0.034	0.003	0.004
γ-tocopherol	rs10466757	BCAT1	T	20	-0.063	0.014	-0.023	0.030	0.016	0.009	-0.022	0.011	-0.015	0.010	-0.015	0.018	-0.002	0.038	0.007	0.005
γ-tocopherol	rs10492212	-	T	20	-0.027	0.006	0.059	0.038	0.019	0.011	-0.005	0.013	0.005	0.012	-0.016	0.020	0.106	0.043	-0.006	0.006
γ-tocopherol	rs10520845	MYO10	A	21	0.191	0.042	-	-	-0.064	0.036	-0.086	0.039	-0.022	0.036	-0.014	0.063	-0.053	0.128	-0.004	0.019
γ-tocopherol	rs1060467	CYP4F11	G	27	-0.023	0.005	-0.058	0.027	-0.009	0.008	-0.015	0.010	0.007	0.009	0.010	0.015	-0.020	0.034	-0.007	0.004
γ-tocopherol	rs13336771	LONP2	A	20	0.062	0.014	-0.039	0.036	-0.008	0.011	-0.013	0.013	-0.011	0.012	0.000	0.020	-0.017	0.044	-0.014	0.006
γ-tocopherol	rs261301	-	C	23	-0.032	0.007	-0.031	0.041	-0.013	0.011	-0.007	0.014	0.005	0.012	0.020	0.021	-0.063	0.049	-0.003	0.006
γ-tocopherol	rs2794327	MEGF6	T	20	-0.036	0.008	-0.016	0.051	0.004	0.010	-0.009	0.011	0.001	0.010	0.002	0.018	-0.033	0.041	-	-
γ-tocopherol	rs5994305	SEC14L2	G	25	-0.031	0.006	0.061	0.034	0.005	0.011	0.007	0.012	-0.003	0.011	-0.022	0.020	0.066	0.044	0.009	0.006
γ-tocopherol	rs6821770	SORCS2	A	20	0.038	0.009	-0.047	0.048	-0.012	0.011	0.008	0.012	0.017	0.011	-0.009	0.019	-0.076	0.046	0.001	0.006

γ-tocopherol	rs7038957	-	C	21	0.029	0.006	0.052	0.037	0.000	0.011	0.035	0.013	0.012	0.012	0.013	0.020	-0.056	0.045	-0.001	0.006
γ-tocopherol	rs7350776	RYR3	G	21	-0.024	0.005	0.003	0.031	0.005	0.009	0.002	0.010	0.016	0.009	0.005	0.016	-0.042	0.037	-0.005	0.005
ascorbate	rs11167905	-	C	24	-0.080	0.016	0.030	0.038	0.005	0.011	0.041	0.013	0.003	0.012	0.001	0.021	-0.043	0.048	0.003	0.006
ascorbate	rs13069990	LSAMP	T	21	-0.051	0.011	0.036	0.027	0.009	0.008	0.007	0.010	0.020	0.009	-0.003	0.015	-0.075	0.035	-0.001	0.004
ascorbate	rs13103690	SLC2A9	G	21	0.047	0.010	0.043	0.029	0.019	0.008	0.004	0.009	0.012	0.009	0.021	0.015	0.029	0.034	0.009	0.005
ascorbate	rs2070006	FGA	C	21	-0.051	0.011	-0.023	0.028	-0.002	0.008	0.000	0.010	0.003	0.009	0.002	0.015	-0.009	0.034	-0.004	0.004
ascorbate	rs577596	-	A	25	-0.057	0.011	-0.011	0.038	-0.006	0.009	0.001	0.011	-0.002	0.010	0.025	0.017	-0.027	0.038	-0.001	0.005
ascorbate	rs6713914	LOC105374754	C	26	-0.059	0.012	-0.042	0.027	-0.006	0.008	-0.005	0.009	0.006	0.009	0.001	0.015	-0.079	0.034	-0.006	0.004
ascorbate	rs6826474	-	T	23	-0.138	0.029	-0.033	0.135	0.028	0.023	0.005	0.027	-0.003	0.024	0.069	0.041	-0.021	0.087	0.018	0.013
ascorbate	rs6834631	LOC107986309	G	24	-0.131	0.027	-0.091	0.133	-0.015	0.019	-0.004	0.023	0.006	0.022	-0.012	0.037	-0.100	0.087	-0.027	0.011
ascorbate	rs7112460	HYLS1	T	24	0.108	0.022	0.070	0.055	0.013	0.016	-0.017	0.019	-0.001	0.018	0.009	0.030	-0.037	0.070	0.011	0.009
ascorbate	rs8057559	ZFHX3	T	20	0.140	0.031	-	-	0.007	0.026	0.047	0.031	-0.014	0.028	0.083	0.046	0.242	0.102	-0.011	0.013
ascorbate	rs808686	-	A	22	0.060	0.013	0.017	0.027	0.011	0.008	-0.001	0.010	0.010	0.009	0.027	0.015	0.067	0.034	0.002	0.004
ascorbate	rs8105491	-	T	22	-0.070	0.015	-0.025	0.037	0.015	0.011	-0.004	0.013	0.021	0.012	0.007	0.021	0.003	0.046	0.007	0.006
ascorbate	rs9419004	ADGRA1	C	20	-0.254	0.056	-0.018	0.061	-0.019	0.014	-0.012	0.013	-0.002	0.011	0.035	0.020	-0.015	0.062	0.004	0.005
ascorbate	rs9606290	LOC105372863	A	20	0.159	0.035	0.028	0.042	0.012	0.011	0.027	0.012	0.016	0.011	0.008	0.019	0.023	0.038	0.008	0.005
retinol	rs10019071	-	A	17	0.657	0.161	-	-	-0.011	0.037	-0.055	0.043	0.025	0.036	0.066	0.076	-0.150	0.159	0.004	0.017
retinol	rs112293959	ZFAND6	G	11	-0.429	0.130	0.043	0.198	-0.005	0.025	0.035	0.030	-0.007	0.029	0.024	0.045	-0.103	0.123	-0.016	0.013
retinol	rs114515641	IGSF11	G	10	0.414	0.129	0.250	0.157	0.030	0.022	-0.029	0.026	0.007	0.024	-0.027	0.041	-0.133	0.094	0.014	0.012
retinol	rs1153379	-	A	15	-0.323	0.083	-0.061	0.128	0.033	0.024	0.005	0.024	0.021	0.023	0.020	0.044	-0.005	0.117	0.023	0.009
retinol	rs117468033	-	T	26	-0.961	0.188	-	-	0.065	0.062	-0.080	0.059	-0.037	0.062	-	-	-	-	-	-
retinol	rs1176744	HTR3B	C	21	-0.207	0.045	0.008	0.028	-0.002	0.008	-0.012	0.010	-0.002	0.009	0.000	0.016	-0.017	0.036	0.005	0.005
retinol	rs118025446	CEP162	A	17	-0.481	0.115	0.137	0.158	-0.011	0.021	-0.001	0.024	0.040	0.022	-0.023	0.037	-0.010	0.087	0.000	0.011
retinol	rs12955464	-	G	15	-0.234	0.061	-0.055	0.068	-0.007	0.013	0.001	0.015	-0.014	0.013	0.032	0.035	0.026	0.059	-0.006	0.006
retinol	rs139726207	-	G	11	0.370	0.110	0.120	0.055	-0.011	0.016	0.015	0.018	0.003	0.017	-0.012	0.032	-0.106	0.063	-0.004	0.009
retinol	rs149113848	-	G	13	-0.964	0.265	-	-	0.037	0.066	-	-	-	-	-	-	-0.161	0.248	0.006	0.026
retinol	rs149478645	CTTNBP2	G	13	-0.507	0.142	-	-	0.017	0.031	-0.050	0.038	0.014	0.033	0.036	0.053	-0.145	0.146	0.002	0.015
retinol	rs17005512	-	C	13	-0.218	0.060	-0.018	0.036	0.017	0.011	-0.011	0.013	-0.003	0.012	-0.013	0.020	0.026	0.045	0.001	0.006
retinol	rs1842947	OR3A3	G	20	-0.192	0.043	0.058	0.031	0.018	0.008	0.019	0.010	-0.008	0.009	-0.009	0.015	0.014	0.035	0.003	0.004

retinol	rs2147337	SIRPG	G	13	0.158	0.044	-0.051	0.028	0.003	0.008	0.004	0.010	-0.027	0.009	0.015	0.016	0.028	0.036	-0.001	0.005
retinol	rs2367816	LOC105374455	G	20	0.228	0.051	0.007	0.031	-0.012	0.009	0.011	0.011	0.004	0.010	0.019	0.017	0.071	0.040	-0.005	0.005
retinol	rs2417325	-	T	15	0.329	0.084	-0.077	0.053	0.005	0.015	0.002	0.018	-0.004	0.016	-0.059	0.028	-0.099	0.064	-0.001	0.008
retinol	rs3890033	TNK2	C	12	0.144	0.042	0.084	0.050	0.005	0.010	-0.001	0.010	0.007	0.009	0.016	0.016	0.053	0.053	0.005	0.005
retinol	rs3898702	LOC101927394	T	16	-0.217	0.054	-0.064	0.034	0.011	0.010	0.015	0.012	-0.010	0.011	0.036	0.020	-0.046	0.043	-0.004	0.005
retinol	rs4135385	CTNNB1	G	19	0.212	0.049	-0.037	0.036	-0.007	0.010	-0.013	0.012	-0.005	0.011	-0.022	0.018	-0.018	0.040	-0.005	0.005
retinol	rs568632536	-	T	14	0.531	0.140	-	-	-	-	-	-	-	-	-	-	-	-	-	-
retinol	rs58411567	DNAH10	A	16	-0.208	0.052	0.073	0.032	0.021	0.009	0.014	0.011	0.023	0.010	0.018	0.017	0.058	0.040	0.019	0.005
retinol	rs6550239	RAB5A	A	15	-0.183	0.048	0.008	0.034	0.002	0.009	0.017	0.011	0.033	0.010	-0.004	0.018	0.045	0.039	0.002	0.005
retinol	rs75308833	-	T	11	-0.494	0.147	-	-	-0.005	0.025	0.026	0.030	-0.019	0.027	0.039	0.046	-0.167	0.113	-0.022	0.014
retinol	rs7926028	-	T	10	-0.135	0.042	-0.126	0.033	-0.008	0.009	0.005	0.010	-0.023	0.009	0.010	0.015	-0.009	0.034	-0.006	0.004
retinol	rs945817	LOC105377858	A	25	-0.275	0.055	-0.025	0.034	0.012	0.010	-0.005	0.012	0.018	0.011	-0.035	0.019	0.017	0.043	0.003	0.005
retinol	rs9586119	-	C	18	0.355	0.083	0.006	0.057	0.002	0.015	0.012	0.017	0.028	0.016	0.021	0.027	0.021	0.063	0.001	0.008
**UK Biobank**																				
α-tocopherol	rs10163969	-	T	19	-0.035	0.008	-0.045	0.039	-0.357	0.169	0.125	0.105	0.001	0.139	0.116	0.268	-0.056	0.265	-	-
α-tocopherol	rs10245705	LOC105375199	T	27	-0.066	0.013	0.050	0.054	-0.104	0.238	-0.124	0.149	-0.035	0.199	0.180	0.380	0.087	0.376	-	-
α-tocopherol	rs10935814	-	A	20	-0.037	0.008	-0.001	0.023	-0.008	0.100	0.034	0.062	-0.051	0.082	-0.127	0.158	-0.245	0.156	-	-
α-tocopherol	rs11145330	-	C	23	-0.032	0.007	0.031	0.023	-0.010	0.102	-0.100	0.064	0.007	0.085	-0.013	0.163	-0.279	0.161	-	-
α-tocopherol	rs11992435	-	G	21	-0.033	0.007	-0.028	0.032	0.092	0.141	-0.051	0.087	-0.044	0.115	0.060	0.223	-0.033	0.221	-	-
α-tocopherol	rs1404410	-	G	21	0.024	0.005	0.012	0.019	0.046	0.081	0.064	0.051	0.045	0.068	0.154	0.130	-0.031	0.128	-	-
α-tocopherol	rs1532701	SLC6A2	A	21	0.014	0.003	-0.001	0.015	-0.114	0.065	0.049	0.041	-0.020	0.054	-0.041	0.104	-0.182	0.102	-	-
α-tocopherol	rs2074731	SF3A1	A	22	-0.018	0.004	0.026	0.020	0.229	0.088	0.087	0.055	-0.009	0.073	0.131	0.140	-0.116	0.139	-	-
α-tocopherol	rs261342	LIPC/LIPC-AS1	C	21	-0.017	0.004	0.002	0.018	0.051	0.079	-0.070	0.049	0.074	0.065	0.098	0.125	0.072	0.124	-	-
α-tocopherol	rs7238006	-	C	24	-0.028	0.006	0.047	0.029	-0.174	0.128	0.025	0.080	0.135	0.106	0.140	0.204	0.025	0.202	-	-
α-tocopherol	rs7930821	ZDHHC13	T	20	0.067	0.015	-0.018	0.053	0.363	0.232	-0.059	0.145	-0.307	0.194	-0.602	0.372	0.186	0.366	-	-
γ-tocopherol	rs10077932	-	T	21	-0.040	0.009	-0.026	0.019	0.031	0.084	-0.011	0.052	-0.061	0.069	-0.050	0.134	-0.015	0.132	-	-
γ-tocopherol	rs1013104	-	T	21	-0.021	0.005	-0.012	0.015	0.119	0.065	-0.069	0.041	-0.096	0.054	-0.229	0.104	-0.019	0.103	-	-
γ-tocopherol	rs10466757	BCAT1	T	20	-0.063	0.014	0.003	0.017	-0.041	0.075	-0.058	0.046	0.046	0.062	-0.174	0.119	0.131	0.117	-	-

γ-tocopherol	rs10492212	-	T	20	-0.027	0.006	-0.012	0.020	0.058	0.089	0.057	0.055	0.019	0.074	-0.062	0.142	0.064	0.139	-	-
γ-tocopherol	rs10520845	MYO10	A	21	0.191	0.042	-0.193	0.069	-0.198	0.301	-0.009	0.188	-0.200	0.251	0.618	0.482	-0.327	0.478	-	-
γ-tocopherol	rs1060467	CYP4F11	G	27	-0.023	0.005	-0.022	0.015	0.147	0.066	0.028	0.041	0.031	0.054	-0.099	0.105	-0.009	0.103	-	-
γ-tocopherol	rs13336771	LONP2	A	20	0.062	0.014	0.010	0.021	-0.157	0.091	0.071	0.056	0.009	0.075	0.148	0.145	0.106	0.143	-	-
γ-tocopherol	rs261301	-	C	23	-0.032	0.007	0.016	0.021	0.012	0.093	-0.004	0.058	0.041	0.077	0.094	0.149	-0.028	0.147	-	-
γ-tocopherol	rs2794327	MEGF6	T	20	-0.036	0.008	0.007	0.016	0.083	0.070	0.014	0.044	-0.101	0.058	0.038	0.112	0.110	0.110	-	-
γ-tocopherol	rs5994305	SEC14L2	G	25	-0.031	0.006	0.018	0.020	0.209	0.087	0.087	0.054	0.012	0.072	0.069	0.138	-0.174	0.137	-	-
γ-tocopherol	rs6821770	SORCS2	A	20	0.038	0.009	0.044	0.019	0.090	0.085	-0.143	0.053	-0.148	0.070	0.037	0.135	0.008	0.134	-	-
γ-tocopherol	rs7038957	-	C	21	0.029	0.006	0.002	0.020	-0.078	0.087	0.002	0.054	-0.021	0.072	0.084	0.138	0.188	0.136	-	-
γ-tocopherol	rs7350776	RYR3	G	21	-0.024	0.005	-0.004	0.016	-0.046	0.071	0.062	0.044	-0.032	0.058	0.082	0.113	-0.011	0.111	-	-
ascorbate	rs11167905	-	C	24	-0.080	0.016	0.039	0.020	0.008	0.090	0.039	0.056	-0.042	0.074	-0.036	0.143	-0.103	0.141	-	-
ascorbate	rs13069990	LSAMP	T	21	-0.051	0.011	-0.016	0.015	0.095	0.066	0.005	0.041	-0.088	0.055	-0.005	0.106	0.091	0.105	-	-
ascorbate	rs13103690	SLC2A9	G	21	0.047	0.010	-0.015	0.015	0.010	0.065	0.026	0.041	0.022	0.054	0.079	0.104	0.181	0.102	-	-
ascorbate	rs2070006	FGA	C	21	-0.051	0.011	0.005	0.015	0.207	0.067	0.019	0.042	0.008	0.055	-0.110	0.107	0.065	0.105	-	-
ascorbate	rs577596	-	A	25	-0.057	0.011	-0.013	0.015	-0.019	0.068	-0.012	0.042	0.128	0.056	0.041	0.108	-0.154	0.107	-	-
ascorbate	rs6713914	LOC105374754	C	26	-0.059	0.012	-0.021	0.015	-0.087	0.065	0.067	0.040	0.066	0.054	0.118	0.104	0.071	0.102	-	-
ascorbate	rs6826474	-	T	23	-0.138	0.029	0.027	0.046	0.003	0.201	-0.040	0.126	-0.130	0.168	0.843	0.325	0.606	0.320	-	-
ascorbate	rs6834631	LOC107986309	G	24	-0.131	0.027	-0.022	0.036	-0.038	0.157	-0.011	0.098	-0.031	0.130	-0.337	0.250	0.294	0.247	-	-
ascorbate	rs7112460	HYLS1	T	24	0.108	0.022	-0.029	0.030	-0.061	0.133	0.102	0.083	-0.003	0.110	0.332	0.212	-0.051	0.209	-	-
ascorbate	rs8057559	ZFHX3	T	20	0.140	0.031	0.026	0.045	0.206	0.198	0.029	0.123	0.145	0.163	-0.044	0.314	-0.071	0.310	-	-
ascorbate	rs808686	-	A	22	0.060	0.013	-0.002	0.015	-0.108	0.065	-0.013	0.040	-0.018	0.054	-0.015	0.103	-0.067	0.102	-	-
ascorbate	rs8105491	-	T	22	-0.070	0.015	-0.016	0.021	0.057	0.090	0.030	0.056	0.071	0.075	-0.152	0.144	-0.110	0.142	-	-
ascorbate	rs9419004	ADGRA1	C	20	-0.254	0.056	-0.013	0.017	0.164	0.073	0.018	0.045	0.057	0.060	0.115	0.116	-0.253	0.114	-	-
ascorbate	rs9606290	LOC105372863	A	20	0.159	0.035	0.000	0.017	0.036	0.075	-0.053	0.047	-0.060	0.062	-0.066	0.119	0.022	0.118	-	-
retinol	rs10019071	-	A	17	0.657	0.161	0.013	0.054	-0.011	0.237	0.010	0.147	0.113	0.195	-0.018	0.377	0.251	0.376	-	-
retinol	rs112293959	ZFAND6	G	11	-0.429	0.130	0.033	0.041	-0.279	0.181	-0.202	0.114	-0.097	0.151	-0.100	0.291	0.037	0.287	-	-
retinol	rs114515641	IGSF11	G	10	0.414	0.129	-0.011	0.043	-0.155	0.188	0.149	0.117	0.150	0.156	0.011	0.299	-0.006	0.295	-	-
retinol	rs1153379	-	A	15	-0.323	0.083	0.024	0.031	-0.201	0.135	0.024	0.084	0.084	0.112	-0.081	0.215	0.305	0.212	-	-
retinol	rs117468033		T	26	-0.961	0.188	-	-	-	-	-	-	-	-	-	-	-	-	-	-

retinol	rs1176744	HTR3B	C	21	-0.207	0.045	0.017	0.016	0.180	0.070	-0.026	0.043	-0.038	0.058	0.204	0.111	-0.159	0.110	-	-
retinol	rs118025446	CEP162	A	17	-0.481	0.115	-0.006	0.038	0.004	0.166	0.024	0.103	0.092	0.136	-0.414	0.263	-0.096	0.260	-	-
retinol	rs12955464	-	G	15	-0.234	0.061	0.020	0.021	0.052	0.092	-0.003	0.057	-0.011	0.076	0.159	0.147	-0.259	0.144	-	-
retinol	rs139726207	-	G	11	0.370	0.110	0.038	0.031	0.206	0.134	0.054	0.084	-0.145	0.111	0.168	0.214	-0.009	0.211	-	-
retinol	rs149113848	-	G	13	-0.964	0.265	0.031	0.086	0.670	0.383	-0.129	0.238	-0.191	0.313	0.779	0.609	-0.312	0.599	-	-
retinol	rs149478645	CTTNBP2	G	13	-0.507	0.142	-0.021	0.050	0.223	0.221	0.073	0.137	-0.208	0.182	0.087	0.351	-0.056	0.346	-	-
retinol	rs17005512	-	C	13	-0.218	0.060	-0.032	0.020	-0.008	0.090	0.044	0.056	-0.052	0.074	0.311	0.143	0.242	0.141	-	-
retinol	rs1842947	OR3A3	G	20	-0.192	0.043	0.011	0.015	0.141	0.065	0.022	0.041	0.034	0.054	0.082	0.104	0.099	0.103	-	-
retinol	rs2147337	SIRPG	G	13	0.158	0.044	0.026	0.015	-0.134	0.068	0.001	0.042	0.045	0.056	0.062	0.108	0.121	0.106	-	-
retinol	rs2367816	LOC105374455	G	20	0.228	0.051	0.003	0.017	-0.042	0.076	0.071	0.047	-0.051	0.063	-0.032	0.121	-0.121	0.120	-	-
retinol	rs2417325	-	T	15	0.329	0.084	-0.044	0.029	-0.111	0.126	0.167	0.079	0.117	0.104	0.201	0.201	0.026	0.198	-	-
retinol	rs3890033	TNK2	C	12	0.144	0.042	-0.004	0.015	-0.056	0.066	-0.065	0.041	0.031	0.054	0.048	0.105	0.011	0.103	-	-
retinol	rs3898702	LOC101927394	T	16	-0.217	0.054	0.002	0.018	-0.065	0.081	-0.015	0.050	0.027	0.067	-0.029	0.129	0.164	0.127	-	-
retinol	rs4135385	CTNNB1	G	19	0.212	0.049	-0.017	0.018	0.043	0.077	-0.025	0.048	0.004	0.064	0.167	0.123	-0.067	0.121	-	-
retinol	rs568632536		T	14	0.531	0.140	-	-	-	-	-	-	-	-	-	-	-	-	-	-
retinol	rs58411567	DNAH10	A	16	-0.208	0.052	0.017	0.018	-0.122	0.077	-0.001	0.048	0.214	0.064	0.063	0.123	0.110	0.122	-	-
retinol	rs6550239	RAB5A	A	15	-0.183	0.048	-0.021	0.018	0.024	0.078	0.034	0.048	0.063	0.064	0.029	0.124	0.244	0.122	-	-
retinol	rs75308833	-	T	11	-0.494	0.147	0.031	0.046	0.498	0.204	0.032	0.127	0.235	0.167	0.313	0.323	0.095	0.321	-	-
retinol	rs7926028	-	T	10	-0.135	0.042	-0.030	0.015	-0.009	0.065	0.019	0.040	0.039	0.054	0.010	0.103	0.028	0.102	-	-
retinol	rs945817	LOC105377858	A	25	-0.275	0.055	0.025	0.019	0.032	0.082	-0.063	0.051	-0.055	0.068	0.093	0.131	-0.232	0.129	-	-
retinol	rs9586119	-	C	18	0.355	0.083	0.029	0.026	0.204	0.115	-0.024	0.072	0.041	0.095	-0.045	0.184	-0.011	0.181	-	-
**FinnGen study**																				
α-tocopherol	rs10163969	-	T	19	-0.035	0.008	0.014	0.017	-	-	0.056	0.032	-0.019	0.043	0.046	0.054	-0.004	0.058	0.027	0.013
α-tocopherol	rs10245705	LOC105375199	T	27	-0.066	0.013	0.065	0.048	-	-	-0.001	0.088	-0.079	0.121	-0.021	0.154	0.021	0.165	0.026	0.036
α-tocopherol	rs10935814	-	A	20	-0.037	0.008	0.035	0.018	-	-	0.009	0.033	0.055	0.045	-0.068	0.056	0.130	0.060	0.005	0.014
α-tocopherol	rs11145330	-	C	23	-0.032	0.007	0.016	0.016	-	-	-0.001	0.029	0.042	0.039	-0.013	0.050	-0.030	0.053	0.006	0.012
α-tocopherol	rs11992435	-	G	21	-0.033	0.007	-0.014	0.027	-	-	-0.067	0.049	0.067	0.067	-0.070	0.085	-0.088	0.091	-0.013	0.020
α-tocopherol	rs1404410	-	G	21	0.024	0.005	-0.007	0.014	-	-	0.036	0.026	-0.043	0.035	-0.021	0.044	-0.007	0.047	0.002	0.011
α-tocopherol	rs1532701	SLC6A2	A	21	0.014	0.003	-0.002	0.011	-	-	-0.018	0.021	-0.070	0.028	-0.027	0.036	0.009	0.038	-0.003	0.009

α-tocopherol	rs2074731	SF3A1	A	22	-0.018	0.004	0.022	0.015	-	-	0.029	0.027	0.032	0.036	0.008	0.046	0.026	0.049	0.018	0.011
α-tocopherol	rs261342	LIPC/LIPC-AS1	C	21	-0.017	0.004	-0.038	0.013	-	-	-0.044	0.025	-0.003	0.034	-0.066	0.042	0.011	0.045	-0.008	0.010
α-tocopherol	rs7238006	-	C	24	-0.028	0.006	0.005	0.016	-	-	-0.010	0.030	0.033	0.041	-0.024	0.051	-0.009	0.055	0.017	0.012
α-tocopherol	rs7930821	ZDHHC13	T	20	0.067	0.015	-0.187	0.059	-	-	-0.127	0.102	-0.209	0.134	-0.246	0.177	-0.067	0.185	-0.044	0.043
γ-tocopherol	rs10077932	-	T	21	-0.040	0.009	0.009	0.014	-	-	0.023	0.026	-0.058	0.036	-0.004	0.045	-0.025	0.048	-0.005	0.011
γ-tocopherol	rs1013104	-	T	21	-0.021	0.005	-0.008	0.011	-	-	-0.013	0.021	0.002	0.028	0.044	0.036	0.070	0.038	-0.005	0.009
γ-tocopherol	rs10466757	BCAT1	T	20	-0.063	0.014	-0.020	0.013	-	-	-0.011	0.024	-0.004	0.032	0.025	0.041	-0.045	0.043	-0.015	0.010
γ-tocopherol	rs10492212	-	T	20	-0.027	0.006	0.005	0.014	-	-	-0.014	0.026	0.005	0.036	-0.026	0.045	0.010	0.048	-0.013	0.011
γ-tocopherol	rs10520845	MYO10	A	21	0.191	0.042	-0.025	0.043	-	-	0.077	0.079	0.089	0.109	0.176	0.135	-0.045	0.141	-0.033	0.032
γ-tocopherol	rs1060467	CYP4F11	G	27	-0.023	0.005	-0.012	0.011	-	-	-0.006	0.021	0.037	0.029	0.003	0.036	0.036	0.038	-0.016	0.009
γ-tocopherol	rs13336771	LONP2	A	20	0.062	0.014	0.006	0.015	-	-	0.022	0.027	0.004	0.038	-0.022	0.047	0.043	0.050	-0.001	0.011
γ-tocopherol	rs261301	-	C	23	-0.032	0.007	0.041	0.015	-	-	0.034	0.028	-0.004	0.038	-0.100	0.047	0.049	0.050	0.010	0.011
γ-tocopherol	rs2794327	MEGF6	T	20	-0.036	0.008	0.022	0.012	-	-	0.014	0.022	0.008	0.030	0.033	0.038	0.014	0.040	0.012	0.009
γ-tocopherol	rs5994305	SEC14L2	G	25	-0.031	0.006	0.017	0.014	-	-	0.020	0.026	0.028	0.036	0.005	0.045	0.029	0.048	0.016	0.011
γ-tocopherol	rs6821770	SORCS2	A	20	0.038	0.009	0.008	0.013	-	-	0.032	0.025	0.000	0.033	0.016	0.042	0.005	0.045	-0.010	0.010
γ-tocopherol	rs7038957	-	C	21	0.029	0.006	0.000	0.015	-	-	0.036	0.028	-0.032	0.039	-0.031	0.049	0.047	0.052	0.006	0.012
γ-tocopherol	rs7350776	RYR3	G	21	-0.024	0.005	-0.003	0.012	-	-	0.005	0.022	-0.027	0.030	0.015	0.038	-0.001	0.040	0.001	0.009
ascorbate	rs11167905	-	C	24	-0.080	0.016	0.003	0.017	-	-	0.035	0.031	-0.036	0.043	0.098	0.054	-0.030	0.057	0.001	0.013
ascorbate	rs13069990	LSAMP	T	21	-0.051	0.011	-0.003	0.012	-	-	-0.009	0.021	0.031	0.029	-0.039	0.037	-0.020	0.039	0.000	0.009
ascorbate	rs13103690	SLC2A9	G	21	0.047	0.010	-0.001	0.011	-	-	0.017	0.021	0.028	0.028	0.069	0.036	0.029	0.038	0.017	0.009
ascorbate	rs2070006	FGA	C	21	-0.051	0.011	-0.009	0.011	-	-	0.010	0.021	-0.023	0.028	0.003	0.035	0.042	0.038	-0.001	0.008
ascorbate	rs577596	-	A	25	-0.057	0.011	0.018	0.011	-	-	-0.007	0.021	-0.011	0.029	0.047	0.036	0.007	0.038	0.010	0.009
ascorbate	rs6713914	LOC105374754	C	26	-0.059	0.012	-0.023	0.011	-	-	0.002	0.021	-0.021	0.029	0.000	0.036	0.032	0.038	-0.012	0.009
ascorbate	rs6826474	-	T	23	-0.138	0.029	0.009	0.041	-	-	0.006	0.076	-0.175	0.105	0.143	0.131	0.173	0.141	0.006	0.031
ascorbate	rs6834631	LOC107986309	G	24	-0.131	0.027	-0.024	0.040	-	-	0.101	0.074	-0.157	0.102	0.080	0.126	-0.031	0.137	-0.010	0.031
ascorbate	rs7112460	HYLS1	T	24	0.108	0.022	-0.054	0.026	-	-	0.017	0.049	0.037	0.067	-0.040	0.084	0.048	0.089	0.007	0.020
ascorbate	rs8057559	ZFHX3	T	20	0.140	0.031	-0.021	0.035	-	-	0.004	0.064	-0.097	0.086	0.281	0.100	-0.053	0.117	-0.013	0.026
ascorbate	rs808686	-	A	22	0.060	0.013	0.011	0.012	-	-	0.009	0.021	-0.014	0.029	0.021	0.036	0.066	0.039	0.002	0.009

ascorbate	rs8105491	-	T	22	-0.070	0.015	-0.011	0.015	-	-	0.022	0.027	-0.011	0.037	-0.031	0.047	-0.009	0.050	-0.009	0.011
ascorbate	rs9419004	ADGRA1	C	20	-0.254	0.056	0.002	0.013	-	-	-0.006	0.024	-0.001	0.033	-0.047	0.041	0.019	0.045	-0.010	0.010
ascorbate	rs9606290	LOC105372863	A	20	0.159	0.035	0.010	0.015	-	-	0.049	0.028	0.044	0.039	0.023	0.048	0.092	0.052	0.020	0.012
retinol	rs10019071	-	A	17	0.657	0.161	-0.032	0.071	-	-	-0.197	0.130	0.037	0.178	0.008	0.218	0.078	0.232	-0.110	0.055
retinol	rs112293959	ZFAND6	G	11	-0.429	0.130	-0.047	0.039	-	-	-0.024	0.071	0.056	0.097	0.216	0.125	-0.057	0.133	-0.002	0.029
retinol	rs114515641	IGSF11	G	10	0.414	0.129	-0.031	0.034	-	-	-0.034	0.063	0.013	0.086	-0.057	0.109	-0.148	0.116	-0.040	0.026
retinol	rs1153379	-	A	15	-0.323	0.083	0.013	0.024	-	-	0.052	0.044	-0.069	0.061	-0.044	0.077	0.001	0.082	0.033	0.018
retinol	rs117468033		T	26	-0.961	0.188	0.013	0.048	-	-	-0.110	0.088	0.205	0.120	-0.138	0.150	0.117	0.161	-0.025	0.037
retinol	rs1176744	HTR3B	C	21	-0.207	0.045	0.002	0.012	-	-	-0.021	0.022	0.032	0.031	-0.020	0.039	0.011	0.041	-0.008	0.009
retinol	rs118025446	CEP162	A	17	-0.481	0.115	0.094	0.040	-	-	-0.014	0.074	0.011	0.102	0.230	0.128	0.031	0.138	0.014	0.030
retinol	rs12955464	-	G	15	-0.234	0.061	0.031	0.017	-	-	0.016	0.031	0.054	0.042	0.136	0.053	0.033	0.056	0.002	0.013
retinol	rs139726207	-	G	11	0.370	0.110	0.021	0.021	-	-	-0.017	0.039	-0.073	0.053	0.000	0.066	0.064	0.071	0.011	0.016
retinol	rs149113848	-	G	13	-0.964	0.265	-0.064	0.092	-	-	-0.147	0.169	-0.197	0.229	-0.088	0.293	-0.246	0.317	-0.076	0.069
retinol	rs149478645	CTTNBP2	G	13	-0.507	0.142	0.003	0.049	-	-	0.016	0.090	0.047	0.123	-0.015	0.153	0.099	0.164	0.052	0.037
retinol	rs17005512	-	C	13	-0.218	0.060	-0.008	0.015	-	-	0.003	0.027	-0.057	0.037	0.037	0.046	-0.016	0.049	0.003	0.011
retinol	rs1842947	OR3A3	G	20	-0.192	0.043	-0.004	0.011	-	-	-0.005	0.021	-0.023	0.028	0.028	0.036	-0.045	0.038	0.013	0.009
retinol	rs2147337	SIRPG	G	13	0.158	0.044	0.008	0.012	-	-	0.009	0.023	0.021	0.031	0.043	0.039	-0.116	0.040	0.012	0.009
retinol	rs2367816	LOC105374455	G	20	0.228	0.051	-0.022	0.013	-	-	-0.029	0.024	0.006	0.033	-0.044	0.042	-0.105	0.045	-0.008	0.010
retinol	rs2417325	-	T	15	0.329	0.084	0.013	0.019	-	-	0.001	0.035	0.025	0.048	-0.051	0.060	0.056	0.064	0.014	0.014
retinol	rs3890033	TNK2	C	12	0.144	0.042	0.013	0.011	-	-	-0.021	0.021	-0.017	0.028	-0.011	0.036	-0.027	0.038	0.005	0.009
retinol	rs3898702	LOC101927394	T	16	-0.217	0.054	0.028	0.013	-	-	0.046	0.025	-0.014	0.033	-0.053	0.042	0.082	0.045	0.025	0.010
retinol	rs4135385	CTNNB1	G	19	0.212	0.049	-0.005	0.014	-	-	-0.020	0.025	-0.001	0.035	-0.031	0.044	0.001	0.047	-0.015	0.010
retinol	rs568632536		T	14	0.531	0.140	-	-	-	-	-	-	-	-	-	-	-	-	-	-
retinol	rs58411567	DNAH10	A	16	-0.208	0.052	0.032	0.013	-	-	0.027	0.024	0.019	0.033	-0.009	0.042	0.031	0.045	0.023	0.010
retinol	rs6550239	RAB5A	A	15	-0.183	0.048	0.042	0.013	-	-	0.015	0.023	0.055	0.032	-0.055	0.040	0.074	0.043	0.021	0.010
retinol	rs75308833	-	T	11	-0.494	0.147	-0.019	0.047	-	-	0.009	0.087	0.038	0.120	-0.186	0.150	0.118	0.158	-0.014	0.035
retinol	rs7926028	-	T	10	-0.135	0.042	0.001	0.011	-	-	0.027	0.021	0.013	0.028	-0.002	0.035	-0.023	0.038	0.016	0.008
retinol	rs945817	LOC105377858	A	25	-0.275	0.055	-0.006	0.018	-	-	0.067	0.033	-0.041	0.045	0.001	0.057	-0.041	0.060	0.000	0.013
retinol	rs9586119	-	C	18	0.355	0.083	-0.011	0.018	-	-	-0.050	0.032	0.008	0.044	-0.057	0.055	-0.032	0.059	-0.020	0.013

Table S6. The MR analysis results of the causal effects of absolute ascorbate levels on major mental disorders.

Outcome	P_h	P_p	MR method	No. SNPs	OR (95% CI)	*P*-value
**Primary outcome**						
**AD**						
ANGST Consortium	0.827	0.854	**Fixed-effect IVW**	**10**	**0.805 (0.574-1.128)**	**0.208**
			Random effect IVW	10	0.805 (0.625-1.038)	0.094
			Maximum likelihood	10	0.807 (0.575-1.132)	0.214
			MR Egger	10	0.843 (0.471-1.509)	0.581
			Weighted median	10	0.781 (0.51-1.196)	0.255
			MR-PRESSO†		NA	NA
UK Biobank	0.929	0.894	**Fixed-effect IVW**	**10**	**0.931 (0.801-1.082)**	**0.351**
			Random effect IVW	10	0.931 (0.845-1.026)	0.147
			Maximum likelihood	10	0.931 (0.8-1.082)	0.350
			MR Egger	10	0.919 (0.726-1.163)	0.502
			Weighted median	10	0.909 (0.754-1.096)	0.316
			MR-PRESSO†		NA	NA
FinnGen	0.431	0.468	**Fixed-effect IVW**	**10**	**1.093 (0.964-1.238)**	**0.165**
			Random effect IVW	10	1.093 (0.964-1.239)	0.167
			Maximum likelihood	10	1.094 (0.965-1.241)	0.161
			MR Egger	10	1.019 (0.817-1.271)	0.872
			Weighted median	10	1.056 (0.899-1.241)	0.507
			MR-PRESSO†		NA	NA
**MDD**						
PGC	0.071	0.059	**Fixed-effect IVW**	**10**	**1.018 (0.936-1.107)**	**0.676**
			Random effect IVW	10	1.018 (0.911-1.137)	0.752
			Maximum likelihood	10	1.019 (0.936-1.108)	0.665
			MR Egger	10	0.899 (0.779-1.039)	0.187
			Weighted median	10	0.962 (0.866-1.068)	0.464
			MR-PRESSO†		NA	NA
UK Biobank	0.040	0.477	Fixed-effect IVW	10	2.263 (1.167-4.39)	0.016
			**Random effect IVW**	**10**	**2.263 (0.896-5.717)**	**0.084**
			Maximum likelihood	10	2.341 (1.196-4.579)	0.013
			MR Egger	10	1.467 (0.333-6.467)	0.626
			Weighted median	10	2.038 (0.856-4.852)	0.107
			MR-PRESSO†		NA	NA
**BIP**						
PGC	<0.001	0.087	Fixed-effect IVW	10	1.093 (0.991-1.206)	0.076
			**Random effect IVW**	**10**	**1.093 (0.862-1.386)**	**0.462**
			Maximum likelihood	10	1.1 (0.993-1.218)	0.067
			MR Egger	10	0.848 (0.611-1.179)	0.356
			Weighted median	10	0.998 (0.88-1.132)	0.975
			MR-PRESSO	9	1.025 (0.927-1.133)	0.633
UK Biobank	0.400	0.158	**Fixed-effect IVW**	**10**	**0.872 (0.577-1.318)**	**0.515**
			Random effect IVW	10	0.872 (0.572-1.33)	0.525
			Maximum likelihood	10	0.871 (0.575-1.319)	0.514
			MR Egger	10	0.588 (0.309-1.121)	0.145

			Weighted median	10	0.679 (0.402-1.146)	0.147
			MR-PRESSO		NA	NA
FinnGen	<0.001	0.111	Fixed-effect IVW	10	1.069 (0.85-1.343)	0.568
			**Random effect IVW**	**10**	**1.069 (0.701-1.629)**	**0.757**
			Maximum likelihood	10	1.073 (0.85-1.355)	0.554
			MR Egger	10	0.661 (0.346-1.262)	0.245
			Weighted median	10	0.703 (0.493-1.004)	0.053
			MR-PRESSO	9	0.962 (0.762-1.215)	0.747
**SCZ**						
PGC	0.003	0.502	Fixed-effect IVW	10	0.925 (0.844-1.014)	0.098
			**Random effect IVW**	**10**	**0.925 (0.793-1.079)**	**0.323**
			Maximum likelihood	10	0.923 (0.841-1.014)	0.094
			MR Egger	10	0.862 (0.669-1.111)	0.284
			Weighted median	10	0.909 (0.807-1.023)	0.113
			MR-PRESSO	9	0.901 (0.788-1.03)	0.127
UK Biobank	0.034	0.585	Fixed-effect IVW	10	1.138 (0.658-1.969)	0.645
			**Random effect IVW**	**10**	**1.138 (0.522-2.478)**	**0.745**
			Maximum likelihood	10	1.14 (0.655-1.984)	0.643
			MR Egger	10	0.858 (0.243-3.039)	0.819
			Weighted median	10	0.908 (0.449-1.837)	0.788
			MR-PRESSO†		NA	NA
FinnGen	0.726	0.804	**Fixed-effect IVW**	**10**	**0.898 (0.655-1.23)**	**0.503**
			Random effect IVW	10	0.898 (0.692-1.165)	0.417
			Maximum likelihood	10	0.897 (0.654-1.231)	0.502
			MR Egger	10	0.847 (0.491-1.461)	0.567
			Weighted median	10	0.861 (0.561-1.32)	0.492
			MR-PRESSO†		NA	NA
**PTSD**						
PGC	0.920	0.294	**Fixed-effect IVW**	**10**	**1.031 (0.882-1.205)**	**0.701**
			Random effect IVW	10	1.031 (0.931-1.142)	0.558
			Maximum likelihood	10	1.031 (0.882-1.206)	0.697
			MR Egger	10	0.925 (0.723-1.182)	0.550
			Weighted median	10	0.958 (0.792-1.16)	0.661
			MR-PRESSO†		NA	NA
UK Biobank	0.776	0.638	**Fixed-effect IVW**	**10**	**2.119 (0.736-6.099)**	**0.164**
			Random effect IVW	10	2.119 (0.918-4.889)	0.078
			Maximum likelihood	10	2.129 (0.736-6.156)	0.163
			MR Egger	10	1.545 (0.296-8.051)	0.620
			Weighted median	10	1.641 (0.435-6.194)	0.465
			MR-PRESSO†		NA	NA
FinnGen	0.117	0.261	**Fixed-effect IVW**	**10**	**0.963 (0.65-1.427)**	**0.851**
			Random effect IVW	10	0.963 (0.588-1.577)	0.881
			Maximum likelihood	10	0.964 (0.648-1.434)	0.858
			MR Egger	10	1.455 (0.639-3.316)	0.398
			Weighted median	10	1.316 (0.718-2.413)	0.374
			MR-PRESSO†		NA	NA

**OCD**						
IOCDF-GC & OCGAS	0.497	0.680	**Fixed-effect IVW**	**10**	**1.131 (0.779-1.641)**	**0.518**
			Random effect IVW	10	1.131 (0.79-1.619)	0.503
			Maximum likelihood	10	1.133 (0.779-1.647)	0.514
			MR Egger	10	1.254 (0.684-2.298)	0.485
			Weighted median	10	1.14 (0.702-1.851)	0.597
			MR-PRESSO†		NA	NA
UK Biobank	0.395	0.310	**Fixed-effect IVW**	**10**	**1.071 (0.378-3.033)**	**0.897**
			Random effect IVW	10	1.071 (0.368-3.115)	0.900
			Maximum likelihood	10	1.073 (0.377-3.057)	0.895
			MR Egger	10	2.159 (0.414-11.245)	0.387
			Weighted median	10	1.401 (0.34-5.783)	0.641
			MR-PRESSO†		NA	NA
FinnGen	0.409	0.844	**Fixed-effect IVW**	**10**	**1.256 (0.827-1.909)**	**0.285**
			Random effect IVW	10	1.256 (0.821-1.923)	0.293
			Maximum likelihood	10	1.26 (0.827-1.919)	0.282
			MR Egger	10	1.178 (0.546-2.54)	0.687
			Weighted median	10	1.264 (0.731-2.185)	0.402
			MR-PRESSO†		NA	NA
**Secondary outcome**						
**Depression**						
PGC & UK Biobank	0.029	0.171	Fixed-effect IVW	10	1.03 (0.985-1.076)	0.196
			**Random effect IVW**	**10**	**1.03 (0.966-1.097)**	**0.368**
			Maximum likelihood	10	1.03 (0.985-1.078)	0.193
			MR Egger	10	0.975 (0.888-1.07)	0.603
			Weighted median	10	1.012 (0.957-1.071)	0.670
			MR-PRESSO†		NA	NA
FinnGen	0.016	0.308	Fixed-effect IVW	10	1.09 (0.992-1.199)	0.073
			**Random effect IVW**	**10**	**1.09 (0.946-1.257)**	**0.232**
			Maximum likelihood	10	1.094 (0.994-1.204)	0.067
			MR Egger	10	0.977 (0.767-1.245)	0.857
			Weighted median	10	1.048 (0.923-1.189)	0.471
			MR-PRESSO	9	1.051 (0.955-1.158)	0.309

The best causal estimation highlighted in bold.

AD, anxiety disorders; MDD, major depressive disorder; BIP, bipolar disorder; SCZ, schizophrenia; PTSD, post-traumatic stress disorder; OCD, obsessive-compulsive disorder; PGC, Psychiatric Genomics Consortium; ANGST, Anxiety NeuroGenetics Study; IOCDF-GC, International Obsessive-Compulsive Disorder Foundation Genetics Collaborative; OCGAS, OCD Collaborative Genetics Association Study. P_h, *P*-value for heterogeneity; P_p, *P*-value for pleiotropy; NA, not applicable;

*: The ORs are scaled per µmol/L increase in ascorbate.

†: No significant outliers.

Table S7. The MR analysis results of the causal effects of absolute retinol levels on major mental disorders.

Outcome	P_h	MR method[§]	No. SNPs	OR (95% CI)	*P*-value
**Primary outcome**					
**AD**					
ANGST Consortium	0.305	**Fixed-effect IVW**	**2**	**0.912 (0.799-1.041)**	**0.171**
		Random effect IVW	2	0.912 (0.796-1.044)	0.182
		Maximum likelihood	2	0.911 (0.797-1.042)	0.174
UK Biobank	0.146	**Fixed-effect IVW**	**2**	**0.99 (0.923-1.062)**	**0.777**
		Random effect IVW	2	0.99 (0.894-1.096)	0.845
		Maximum likelihood	2	0.99 (0.922-1.062)	0.775
FinnGen	0.002	Fixed-effect IVW	2	0.979 (0.926-1.034)	0.445
		**Random effect IVW**	**2**	**0.979 (0.827-1.158)**	**0.802**
		Maximum likelihood	2	0.977 (0.922-1.035)	0.426
**MDD**					
PGC	0.101	**Fixed-effect IVW**	**2**	**1.026 (0.988-1.065)**	**0.183**
		Random effect IVW	2	1.026 (0.964-1.091)	0.417
		Maximum likelihood	2	1.027 (0.988-1.067)	0.182
UK Biobank	0.748	**Fixed-effect IVW**	**2**	**0.811 (0.596-1.103)**	**0.182**
		Random effect IVW	2	0.811 (0.735-0.895)	<0.001
		Maximum likelihood	2	0.811 (0.595-1.106)	0.186
**BIP**					
PGC	0.387	**Fixed-effect IVW**	**2**	**0.993 (0.949-1.039)**	**0.768**
		Random effect IVW	2	0.993 (0.955-1.033)	0.733
		Maximum likelihood	2	0.993 (0.949-1.039)	0.767
UK Biobank	0.123	**Fixed-effect IVW**	**2**	**0.877 (0.724-1.063)**	**0.181**
		Random effect IVW	2	0.877 (0.653-1.179)	0.386
		Maximum likelihood	2	0.875 (0.72-1.064)	0.180
FinnGen	0.839	**Fixed-effect IVW**	**2**	**1.035 (0.935-1.145)**	**0.511**
		Random effect IVW	2	1.035 (1.013-1.056)	0.001
		Maximum likelihood	2	1.035 (0.935-1.145)	0.512
**SCZ**					
PGC	0.993	**Fixed-effect IVW**	**2**	**1.039 (0.999-1.081)**	**0.054**
		Random effect IVW	2	1.039 (1.039-1.04)	<0.001
		Maximum likelihood	2	1.039 (0.999-1.081)	0.058
UK Biobank	0.385	**Fixed-effect IVW**	**2**	**0.977 (0.757-1.259)**	**0.855**
		Random effect IVW	2	0.977 (0.783-1.218)	0.833
		Maximum likelihood	2	0.976 (0.756-1.26)	0.854
FinnGen	0.012	Fixed-effect IVW	2	0.969 (0.843-1.114)	0.657
		**Random effect IVW**	**2**	**0.969 (0.682-1.376)**	**0.860**
		Maximum likelihood	2	0.967 (0.838-1.116)	0.648
**PTSD**					
PGC	0.484	**Fixed-effect IVW**	**2**	**1.054 (0.983-1.13)**	**0.142**
		Random effect IVW	2	1.054 (1.004-1.107)	0.036
		Maximum likelihood	2	1.054 (0.982-1.132)	0.145
UK Biobank	0.838	**Fixed-effect IVW**	**2**	**1.421 (0.87-2.32)**	**0.160**
		Random effect IVW	2	1.421 (1.285-1.571)	<0.001

		Maximum likelihood	2	1.421 (0.866-2.331)	0.164
FinnGen	0.262	**Fixed-effect IVW**	**2**	**0.914 (0.767-1.088)**	**0.312**
		Random effect IVW	2	0.914 (0.751-1.112)	0.368
		Maximum likelihood	2	0.913 (0.765-1.089)	0.311
**OCD**					
IOCDF-GC & OCGAS	0.536	**Fixed-effect IVW**	**2**	**1.087 (0.928-1.273)**	**0.303**
		Random effect IVW	2	1.087 (0.985-1.199)	0.096
		Maximum likelihood	2	1.087 (0.927-1.275)	0.304
UK Biobank	0.332	**Fixed-effect IVW**	**2**	**0.888 (0.547-1.44)**	**0.629**
		Random effect IVW	2	0.888 (0.555-1.419)	0.619
		Maximum likelihood	2	0.887 (0.546-1.442)	0.628
FinnGen	0.984	**Fixed-effect IVW**	**2**	**0.959 (0.796-1.156)**	**0.662**
		Random effect IVW	2	0.959 (0.956-0.963)	<0.001
		Maximum likelihood	2	0.959 (0.796-1.156)	0.663
**Secondary outcome**					
**Depression**					
PGC & UK Biobank	0.009	Fixed-effect IVW	2	1.008 (0.988-1.029)	0.450
		**Random effect IVW**	**2**	**1.008 (0.956-1.063)**	**0.773**
		Maximum likelihood	2	1.008 (0.987-1.03)	0.437
FinnGen	0.092	**Fixed-effect IVW**	**2**	**1.038 (0.996-1.083)**	**0.079**
		Random effect IVW	2	1.038 (0.968-1.114)	0.296
		Maximum likelihood	2	1.039 (0.995-1.085)	0.080

The best causal estimation highlighted in bold.

AD, anxiety disorders; MDD, major depressive disorder; BIP, bipolar disorder; SCZ, schizophrenia; PTSD, post-traumatic stress disorder; OCD, obsessive-compulsive disorder; PGC, Psychiatric Genomics Consortium; ANGST, Anxiety NeuroGenetics Study; IOCDF-GC, International Obsessive-Compulsive Disorder Foundation Genetics Collaborative; OCGAS, OCD Collaborative Genetics Association Study. P_h, P-value for heterogeneity.

*: The ORs are scaled per 0.1 unit increase in ln-transformed retinol.

§: Since the number of instrumental variable SNPs was less than 3, the weighted median, MR-Egger and MR-PRESSO were not included in the analysis method.

Table S8. The MR analysis results of the causal effects of absolute β-carotene levels on major mental disorders.

Outcome	P_h	MR method§	No. SNPs	OR (95% CI)	*P*-value
**Primary outcome**					
**AD**					
ANGST Consortium	0.604	**Fixed-effect IVW**	**2**	**1.348 (0.968-1.878)**	**0.077**
		Random effect IVW	2	1.348 (1.135-1.601)	0.001
		Maximum likelihood	2	1.348 (0.964-1.886)	0.081
UK Biobank	0.349	**Fixed-effect IVW**	**2**	**1.136 (0.947-1.361)**	**0.169**
		Random effect IVW	2	1.136 (0.958-1.345)	0.142
		Maximum likelihood	2	1.136 (0.946-1.365)	0.171
FinnGen	0.987	**Fixed-effect IVW**	**2**	**0.997 (0.867-1.147)**	**0.971**
		Random effect IVW	2	0.997 (0.995-1)	0.025
		Maximum likelihood	2	0.997 (0.867-1.147)	0.971
**MDD**					
PGC	0.091	**Fixed-effect IVW**	**2**	**1.004 (0.911-1.108)**	**0.931**
		Random effect IVW	2	1.004 (0.851-1.185)	0.959
		Maximum likelihood	2	1.004 (0.91-1.109)	0.930
UK Biobank	0.803	**Fixed-effect IVW**	**2**	**1.408 (0.635-3.124)**	**0.400**
		Random effect IVW	2	1.408 (1.155-1.718)	0.001
		Maximum likelihood	2	1.409 (0.633-3.132)	0.401
**BIP**					
PGC	0.953	**Fixed-effect IVW**	**2**	**0.946 (0.843-1.062)**	**0.349**
		Random effect IVW	2	0.946 (0.94-0.953)	<0.001
		Maximum likelihood	2	0.946 (0.843-1.062)	0.351
UK Biobank	0.524	**Fixed-effect IVW**	**2**	**0.733 (0.446-1.205)**	**0.221**
		Random effect IVW	2	0.733 (0.534-1.007)	0.055
		Maximum likelihood	2	0.733 (0.444-1.209)	0.223
FinnGen	0.583	**Fixed-effect IVW**	**2**	**0.926 (0.717-1.196)**	**0.556**
		Random effect IVW	2	0.926 (0.805-1.066)	0.284
		Maximum likelihood	2	0.926 (0.717-1.196)	0.556
**SCZ**					
PGC	0.926	**Fixed-effect IVW**	**2**	**1.018 (0.915-1.132)**	**0.748**
		Random effect IVW	2	1.018 (1.008-1.028)	0.001
		Maximum likelihood	2	1.018 (0.915-1.132)	0.748
UK Biobank	0.337	**Fixed-effect IVW**	**2**	**0.743 (0.384-1.436)**	**0.377**
		Random effect IVW	2	0.743 (0.395-1.398)	0.357
		Maximum likelihood	2	0.741 (0.382-1.44)	0.377
FinnGen	0.570	**Fixed-effect IVW**	**2**	**1.261 (0.89-1.787)**	**0.191**
		Random effect IVW	2	1.261 (1.035-1.537)	0.022
		Maximum likelihood	2	1.262 (0.888-1.793)	0.194
**PTSD**					
PGC	0.656	**Fixed-effect IVW**	**2**	**0.919 (0.767-1.1)**	**0.357**
		Random effect IVW	2	0.919 (0.848-0.996)	0.038
		Maximum likelihood	2	0.919 (0.766-1.101)	0.359
UK Biobank	0.832	**Fixed-effect IVW**	**2**	**1.131 (0.317-4.033)**	**0.849**
		Random effect IVW	2	1.131 (0.864-1.482)	0.370

		Maximum likelihood	2	1.132 (0.317-4.035)	0.849
FinnGen	0.881	**Fixed-effect IVW**	**2**	**1.347 (0.867-2.093)**	**0.184**
		Random effect IVW	2	1.347 (1.261-1.44)	<0.001
		Maximum likelihood	2	1.348 (0.865-2.1)	0.188
**OCD**					
IOCDF-GC & OCGAS	0.246	**Fixed-effect IVW**	**2**	**0.83 (0.546-1.261)**	**0.382**
		Random effect IVW	2	0.83 (0.511-1.348)	0.451
		Maximum likelihood	2	0.828 (0.544-1.263)	0.381
UK Biobank	0.222	**Fixed-effect IVW**	**2**	**1.522 (0.435-5.326)**	**0.511**
		Random effect IVW	2	1.522 (0.329-7.037)	0.591
		Maximum likelihood	2	1.529 (0.432-5.409)	0.510
FinnGen	0.081	**Fixed-effect IVW**	**2**	**0.947 (0.593-1.514)**	**0.821**
		Random effect IVW	2	0.947 (0.418-2.149)	0.897
		Maximum likelihood	2	0.946 (0.588-1.522)	0.819
**Secondary outcome**					
**Depression**					
PGC	0.079	**Fixed-effect IVW**	**2**	**1.004 (0.952-1.059)**	**0.879**
		Random effect IVW	2	1.004 (0.915-1.102)	0.931
		Maximum likelihood	2	1.004 (0.952-1.059)	0.878
FinnGen	0.461	**Fixed-effect IVW**	**2**	**0.911 (0.82-1.013)**	**0.084**
		Random effect IVW	2	0.911 (0.843-0.985)	0.019
		Maximum likelihood	2	0.911 (0.818-1.014)	0.087

The best causal estimation highlighted in bold.

AD, anxiety disorders; MDD, major depressive disorder; BIP, bipolar disorder; SCZ, schizophrenia; PTSD, post-traumatic stress disorder; OCD, obsessive-compulsive disorder; PGC, Psychiatric Genomics Consortium; ANGST, Anxiety NeuroGenetics Study; IOCDF-GC, International Obsessive-Compulsive Disorder Foundation Genetics Collaborative; OCGAS, OCD Collaborative Genetics Association Study. P_h, P-value for heterogeneity.

*: The ORs are scaled per unit increase in ln-transformed β-carotene.

§: Since the number of instrumental variable SNPs was less than 3, the weighted median, MR-Egger and MR-PRESSO were not included in the analysis method.

Table S9. The MR analysis results of the causal effects of absolute lycopene levels on major mental disorders.

Outcome	P_h	P_p	MR method	No. SNPs	OR (95% CI)	*P*-value
**Primary outcome**						
**AD**						
ANGST Consortium	0.210	0.205	**Fixed-effect IVW**	**4**	**0.968 (0.881-1.063)**	**0.494**
			Random effect IVW	4	0.968 (0.862-1.086)	0.577
			Maximum likelihood	4	0.967 (0.881-1.063)	0.489
			MR Egger	4	0.834 (0.694-1.002)	0.192
			Weighted median	4	0.934 (0.833-1.047)	0.240
			MR-PRESSO†		NA	NA
UK Biobank	0.964	0.612	**Fixed-effect IVW**	**5**	**1.038 (0.977-1.102)**	**0.227**
			Random effect IVW	5	1.038 (1.014-1.062)	0.002
			Maximum likelihood	5	1.038 (0.977-1.103)	0.228
			MR Egger	5	1.066 (0.953-1.193)	0.342
			Weighted median	5	1.047 (0.976-1.124)	0.200
			MR-PRESSO†		NA	NA
FinnGen	0.897	0.627	**Fixed-effect IVW**	**5**	**0.939 (0.894-0.987)**	**0.013**
			Random effect IVW	5	0.939 (0.902-0.977)	0.002
			Maximum likelihood	5	0.937 (0.889-0.989)	0.018
			MR Egger	5	0.934 (0.855-1.021)	0.228
			Weighted median	5	0.947 (0.892-1.006)	0.075
			MR-PRESSO†		NA	NA
**MDD**						
PGC	0.013	0.082	Fixed-effect IVW	5	1.033 (0.997-1.069)	0.071
			**Random effect IVW**	**5**	**1.033 (0.971-1.099)**	**0.310**
			Maximum likelihood	5	1.037 (0.998-1.077)	0.066
			MR Egger	5	0.95 (0.881-1.024)	0.272
			Weighted median	5	1.01 (0.962-1.06)	0.689
			MR-PRESSO†		NA	NA
UK Biobank	0.626	0.380	**Fixed-effect IVW**	**5**	**1.251 (0.959-1.63)**	**0.098**
			Random effect IVW	5	1.251 (1.01-1.549)	0.041
			Maximum likelihood	5	1.256 (0.962-1.639)	0.094
			MR Egger	5	1.554 (0.95-2.542)	0.177
			Weighted median	5	1.101 (0.79-1.533)	0.570
			MR-PRESSO†		NA	NA
**BIP**						
PGC	0.465	0.374	**Fixed-effect IVW**	**5**	**1.005 (0.965-1.047)**	**0.813**
			Random effect IVW	5	1.005 (0.967-1.045)	0.803
			Maximum likelihood	5	1.005 (0.964-1.048)	0.809
			MR Egger	5	1.039 (0.964-1.12)	0.389
			Weighted median	5	1.012 (0.957-1.07)	0.671
			MR-PRESSO†		NA	NA
UK Biobank	0.330	0.855	**Fixed-effect IVW**	**5**	**0.922 (0.782-1.088)**	**0.338**
			Random effect IVW	5	0.922 (0.772-1.102)	0.372
			Maximum likelihood	5	0.919 (0.774-1.091)	0.332
			MR Egger	5	0.953 (0.653-1.391)	0.818

			Weighted median	5	0.979 (0.796-1.203)	0.838
			MR-PRESSO†		NA	NA
FinnGen	0.836	0.597	**Fixed-effect IVW**	**5**	**0.975 (0.89-1.068)**	**0.587**
			Random effect IVW	5	0.975 (0.923-1.03)	0.366
			Maximum likelihood	5	0.975 (0.889-1.069)	0.587
			MR Egger	5	1.015 (0.863-1.195)	0.865
			Weighted median	5	0.985 (0.881-1.1)	0.784
			MR-PRESSO†		NA	NA
**SCZ**						
PGC	0.212	0.158	**Fixed-effect IVW**	**5**	**1.003 (0.966-1.04)**	**0.882**
			Random effect IVW	5	1.003 (0.959-1.048)	0.902
			Maximum likelihood	5	1.003 (0.966-1.042)	0.880
			MR Egger	5	0.95 (0.887-1.016)	0.232
			Weighted median	5	0.978 (0.935-1.023)	0.337
			MR-PRESSO†		NA	NA
UK Biobank	0.370	0.950	**Fixed-effect IVW**	**5**	**0.982 (0.788-1.223)**	**0.869**
			Random effect IVW	5	0.982 (0.782-1.232)	0.873
			Maximum likelihood	5	0.981 (0.784-1.227)	0.866
			MR Egger	5	0.968 (0.594-1.577)	0.904
			Weighted median	5	1.048 (0.794-1.384)	0.741
			MR-PRESSO†		NA	NA
FinnGen	0.084	0.708	**Fixed-effect IVW**	**5**	**1.075 (0.948-1.219)**	**0.257**
			Random effect IVW	5	1.075 (0.898-1.287)	0.430
			Maximum likelihood	5	1.078 (0.948-1.227)	0.251
			MR Egger	5	1.145 (0.797-1.645)	0.516
			Weighted median	5	1.074 (0.919-1.256)	0.368
			MR-PRESSO†		NA	NA
**PTSD**						
PGC	0.986	0.797	**Fixed-effect IVW**	**5**	**1.001 (0.937-1.069)**	**0.969**
			Random effect IVW	5	1.001 (0.982-1.021)	0.897
			Maximum likelihood	5	1.001 (0.937-1.07)	0.969
			MR Egger	5	0.987 (0.873-1.115)	0.842
			Weighted median	5	1.007 (0.933-1.087)	0.853
			MR-PRESSO†		NA	NA
UK Biobank	0.782	0.526	**Fixed-effect IVW**	**5**	**1.111 (0.728-1.697)**	**0.625**
			Random effect IVW	5	1.111 (0.84-1.47)	0.460
			Maximum likelihood	5	1.113 (0.728-1.703)	0.622
			MR Egger	5	1.416 (0.645-3.108)	0.450
			Weighted median	5	1.063 (0.636-1.776)	0.816
			MR-PRESSO†		NA	NA
FinnGen	0.597	0.670	**Fixed-effect IVW**	**5**	**1.027 (0.878-1.202)**	**0.738**
			Random effect IVW	5	1.027 (0.901-1.171)	0.688
			Maximum likelihood	5	1.027 (0.877-1.204)	0.738
			MR Egger	5	1.086 (0.82-1.44)	0.604
			Weighted median	5	1.023 (0.846-1.238)	0.812
			MR-PRESSO†		NA	NA

**OCD**						
IOCDF-GC & OCGAS	0.367	0.603	**Fixed-effect IVW**	**5**	**1.043 (0.895-1.216)**	**0.588**
			Random effect IVW	5	1.043 (0.89-1.223)	0.601
			Maximum likelihood	5	1.044 (0.895-1.218)	0.581
			MR Egger	5	1.132 (0.817-1.57)	0.510
			Weighted median	5	1.092 (0.899-1.326)	0.376
			MR-PRESSO†		NA	NA
UK Biobank	0.279	0.829	**Fixed-effect IVW**	**5**	**0.794 (0.523-1.205)**	**0.278**
			Random effect IVW	5	0.794 (0.496-1.271)	0.336
			Maximum likelihood	5	0.788 (0.514-1.206)	0.272
			MR Egger	5	0.717 (0.264-1.95)	0.561
			Weighted median	5	0.742 (0.427-1.289)	0.290
			MR-PRESSO†		NA	NA
FinnGen	0.730	0.450	**Fixed-effect IVW**	**5**	**0.926 (0.783-1.094)**	**0.364**
			Random effect IVW	5	0.926 (0.822-1.043)	0.203
			Maximum likelihood	5	0.924 (0.778-1.097)	0.367
			MR Egger	5	1.033 (0.766-1.392)	0.847
			Weighted median	5	0.974 (0.798-1.189)	0.799
			MR-PRESSO†		NA	NA
**Secondary outcome**						
**Depression**						
PGC & UK Biobank	0.307	0.200	**Fixed-effect IVW**	**5**	**1.004 (0.986-1.022)**	**0.693**
			Random effect IVW	5	1.004 (0.984-1.024)	0.719
			Maximum likelihood	5	1.004 (0.985-1.022)	0.688
			MR Egger	5	0.98 (0.948-1.014)	0.328
			Weighted median	5	1.002 (0.98-1.026)	0.837
			MR-PRESSO†		NA	NA
FinnGen	0.265	0.307	**Fixed-effect IVW**	**5**	**0.973 (0.938-1.011)**	**0.158**
			Random effect IVW	5	0.973 (0.933-1.016)	0.217
			Maximum likelihood	5	0.972 (0.933-1.012)	0.166
			MR Egger	5	1.01 (0.94-1.086)	0.796
			Weighted median	5	0.996 (0.947-1.046)	0.860
			MR-PRESSO†		NA	NA

The best causal estimation highlighted in bold.

AD, anxiety disorders; MDD, major depressive disorder; BIP, bipolar disorder; SCZ, schizophrenia; PTSD, post-traumatic stress disorder; OCD, obsessive-compulsive disorder; PGC, Psychiatric Genomics Consortium; ANGST, Anxiety NeuroGenetics Study; IOCDF-GC, International Obsessive-Compulsive Disorder Foundation Genetics Collaborative; OCGAS, OCD Collaborative Genetics Association Study. P_h, *P*-value for heterogeneity; P_p, *P*-value for pleiotropy; NA, not applicable;

*: The ORs are scaled per µg/dL increase in lycopene.

†: No significant outliers.

Table S10. The MR analysis results of the causal effects of α-tocopherol metabolites on major mental disorders.

Outcome	P_h	P_p	MR method	No. SNPs	OR (95% CI)	*P*-value
**Primary outcome**						
**AD**						
ANGST Consortium	0.878	0.329	**Fixed-effect IVW**	**9**	**0.995 (0.893-1.11)**	**0.933**
			Random effect IVW	9	0.995 (0.924-1.072)	0.902
			Maximum likelihood	9	0.995 (0.892-1.111)	0.932
			MR Egger	9	1.175 (0.846-1.63)	0.368
			Weighted median	9	1.039 (0.9-1.2)	0.598
			MR-PRESSO†		NA	NA
UK Biobank	0.558	0.986	**Fixed-effect IVW**	**11**	**0.977 (0.928-1.028)**	**0.363**
			Random effect IVW	11	0.977 (0.931-1.024)	0.330
			Maximum likelihood	11	0.976 (0.926-1.028)	0.357
			MR Egger	11	0.976 (0.864-1.101)	0.698
			Weighted median	11	0.99 (0.923-1.062)	0.788
			MR-PRESSO†		NA	NA
FinnGen	0.011	0.086	Fixed-effect IVW	11	0.956 (0.921-0.993)	0.018
			**Random effect IVW**	**11**	**0.956 (0.904-1.012)**	**0.120**
			Maximum likelihood	11	0.953 (0.917-0.991)	0.016
			MR Egger	11	0.848 (0.742-0.968)	0.037
			Weighted median	11	0.958 (0.91-1.008)	0.101
			MR-PRESSO	10	0.967 (0.931-1.005)	0.084
**MDD**						
PGC	0.519	0.392	**Fixed-effect IVW**	**11**	**0.999 (0.973-1.027)**	**0.963**
			Random effect IVW	11	0.999 (0.974-1.026)	0.961
			Maximum likelihood	11	0.999 (0.972-1.027)	0.962
			MR Egger	11	0.973 (0.912-1.038)	0.425
			Weighted median	11	1.009 (0.974-1.044)	0.619
			MR-PRESSO†		NA	NA
UK Biobank	0.031	0.052	Fixed-effect IVW	11	1.045 (0.835-1.308)	0.702
			**Random effect IVW**	**11**	**1.045 (0.762-1.433)**	**0.786**
			Maximum likelihood	11	1.048 (0.831-1.321)	0.693
			MR Egger	11	2.146 (1.183-3.892)	0.033
			Weighted median	11	1.033 (0.76-1.405)	0.835
			MR-PRESSO†		NA	NA
**BIP**						
PGC	0.680	0.932	**Fixed-effect IVW**	**11**	**1.015 (0.984-1.048)**	**0.346**
			Random effect IVW	11	1.015 (0.988-1.043)	0.276
			Maximum likelihood	11	1.016 (0.984-1.049)	0.340
			MR Egger	11	1.012 (0.938-1.092)	0.763
			Weighted median	11	1.016 (0.974-1.059)	0.459
			MR-PRESSO†		NA	NA
UK Biobank	0.272	0.429	**Fixed-effect IVW**	**11**	**1.069 (0.93-1.23)**	**0.349**
			Random effect IVW	11	1.069 (0.916-1.248)	0.397
			Maximum likelihood	11	1.074 (0.93-1.241)	0.330
			MR Egger	11	0.927 (0.638-1.346)	0.699

			Weighted median	11	1.129 (0.927-1.376)	0.228
			MR-PRESSO†		NA	NA
FinnGen	0.184	0.546	**Fixed-effect IVW**	**11**	**0.992 (0.927-1.062)**	**0.825**
			Random effect IVW	11	0.992 (0.916-1.075)	0.850
			Maximum likelihood	11	0.992 (0.925-1.064)	0.827
			MR Egger	11	0.93 (0.748-1.156)	0.532
			Weighted median	11	1.001 (0.911-1.1)	0.979
			MR-PRESSO†		NA	NA
**SCZ**						
PGC	0.124	0.172	**Fixed-effect IVW**	**11**	**0.992 (0.964-1.022)**	**0.608**
			Random effect IVW	11	0.992 (0.957-1.029)	0.678
			Maximum likelihood	11	0.992 (0.963-1.023)	0.614
			MR Egger	11	0.938 (0.865-1.018)	0.160
			Weighted median	11	0.981 (0.941-1.023)	0.374
			MR-PRESSO†		NA	NA
UK Biobank	0.793	0.898	**Fixed-effect IVW**	**11**	**0.948 (0.788-1.142)**	**0.576**
			Random effect IVW	11	0.948 (0.819-1.098)	0.479
			Maximum likelihood	11	0.947 (0.784-1.144)	0.572
			MR Egger	11	0.974 (0.627-1.515)	0.911
			Weighted median	11	1.051 (0.818-1.351)	0.696
			MR-PRESSO†		NA	NA
FinnGen	0.522	0.216	**Fixed-effect IVW**	**11**	**0.886 (0.808-0.972)**	**0.011**
			Random effect IVW	11	0.886 (0.811-0.968)	0.008
			Maximum likelihood	11	0.883 (0.801-0.972)	0.011
			MR Egger	11	1.033 (0.81-1.319)	0.798
			Weighted median	11	0.875 (0.772-0.993)	0.039
			MR-PRESSO†		NA	NA
**PTSD**						
PGC	0.567	0.851	**Fixed-effect IVW**	**11**	**1.024 (0.973-1.077)**	**0.362**
			Random effect IVW	11	1.024 (0.977-1.073)	0.327
			Maximum likelihood	11	1.025 (0.973-1.079)	0.355
			MR Egger	11	1.013 (0.898-1.143)	0.841
			Weighted median	11	1.038 (0.97-1.11)	0.285
			MR-PRESSO†		NA	NA
UK Biobank	0.758	0.953	**Fixed-effect IVW**	**11**	**0.868 (0.607-1.241)**	**0.438**
			Random effect IVW	11	0.868 (0.648-1.162)	0.341
			Maximum likelihood	11	0.863 (0.598-1.247)	0.433
			MR Egger	11	0.847 (0.362-1.982)	0.711
			Weighted median	11	0.802 (0.489-1.314)	0.381
			MR-PRESSO†		NA	NA
FinnGen	0.603	0.826	**Fixed-effect IVW**	**11**	**1.018 (0.906-1.145)**	**0.761**
			Random effect IVW	11	1.018 (0.916-1.133)	0.738
			Maximum likelihood	11	1.018 (0.904-1.147)	0.764
			MR Egger	11	0.985 (0.723-1.343)	0.927
			Weighted median	11	1.036 (0.886-1.211)	0.660
			MR-PRESSO†		NA	NA

**OCD**						
IOCDF-GC & OCGAS	0.157	0.906	**Fixed-effect IVW**	**11**	**0.927 (0.825-1.04)**	**0.198**
			Random effect IVW	11	0.927 (0.807-1.065)	0.283
			Maximum likelihood	11	0.922 (0.818-1.04)	0.187
			MR Egger	11	0.946 (0.663-1.349)	0.765
			Weighted median	11	0.961 (0.813-1.136)	0.642
			MR-PRESSO†		NA	NA
UK Biobank	0.518	0.136	**Fixed-effect IVW**	**11**	**1.196 (0.84-1.703)**	**0.320**
			Random effect IVW	11	1.196 (0.853-1.677)	0.298
			Maximum likelihood	11	1.212 (0.846-1.737)	0.293
			MR Egger	11	2.258 (0.977-5.219)	0.089
			Weighted median	11	1.163 (0.735-1.843)	0.519
			MR-PRESSO†		NA	NA
IOCDF-GC & OCGAS	0.803	0.737	**Fixed-effect IVW**	**11**	**0.962 (0.849-1.09)**	**0.540**
			Random effect IVW	11	0.962 (0.872-1.061)	0.435
			Maximum likelihood	11	0.96 (0.846-1.091)	0.535
			MR Egger	11	0.911 (0.655-1.268)	0.594
			Weighted median	11	0.996 (0.844-1.176)	0.963
			MR-PRESSO†		NA	NA
**Secondary outcome**						
**Depression**						
PGC & UK Biobank	0.916	0.608	**Fixed-effect IVW**	**11**	**0.996 (0.981-1.011)**	**0.568**
			Random effect IVW	11	0.996 (0.986-1.006)	0.400
			Maximum likelihood	11	0.996 (0.981-1.011)	0.565
			MR Egger	11	1.004 (0.97-1.04)	0.815
			Weighted median	11	0.999 (0.98-1.019)	0.920
			MR-PRESSO†		NA	NA
FinnGen	0.629	0.569	**Fixed-effect IVW**	**11**	**0.97 (0.943-0.998)**	**0.035**
			Random effect IVW	11	0.97 (0.946-0.995)	0.018
			Maximum likelihood	11	0.97 (0.942-0.998)	0.036
			MR Egger	11	0.95 (0.882-1.024)	0.212
			Weighted median	11	0.98 (0.943-1.019)	0.314
			MR-PRESSO†		NA	NA

The best causal estimation highlighted in bold.

AD, anxiety disorders; MDD, major depressive disorder; BIP, bipolar disorder; SCZ, schizophrenia; PTSD, post-traumatic stress disorder; OCD, obsessive-compulsive disorder; PGC, Psychiatric Genomics Consortium; ANGST, Anxiety NeuroGenetics Study; IOCDF-GC, International Obsessive-Compulsive Disorder Foundation Genetics Collaborative; OCGAS, OCD Collaborative Genetics Association Study. P_h, P-value for heterogeneity; P_p, P-value for pleiotropy; NA, not applicable;

*: The ORs are scaled per 0.1 unit increase in log-transformed α-tocopherol.

†: No significant outliers.

Table S11. The MR analysis results of the causal effects of γ-tocopherol metabolites on major mental disorders.

Outcome	P_h	P_p	MR method	No. SNPs	OR (95% CI)	*P*-value
**Primary outcome**						
**AD**						
ANGST Consortium	0.042	0.290	Fixed-effect IVW	12	1.004 (0.953-1.058)	0.884
			**Random effect IVW**	**12**	**1.004 (0.935-1.078)**	**0.914**
			Maximum likelihood	12	1.004 (0.951-1.06)	0.881
			MR Egger	12	0.915 (0.766-1.093)	0.349
			Weighted median	12	1.001 (0.925-1.084)	0.978
			MR-PRESSO†		NA	NA
UK Biobank	0.066	0.048	Fixed-effect IVW	13	1.003 (0.978-1.029)	0.822
			Random effect IVW	13	1.003 (0.97-1.036)	0.862
			Maximum likelihood	13	1.003 (0.977-1.03)	0.815
			**MR Egger**	**13**	**0.941 (0.883-1.002)**	**0.084**
			Weighted median	13	1.002 (0.966-1.04)	0.902
			MR-PRESSO†		NA	NA
FinnGen	0.122	0.770	**Fixed-effect IVW**	**13**	**0.997 (0.979-1.015)**	**0.722**
			Random effect IVW	13	0.997 (0.975-1.019)	0.770
			Maximum likelihood	13	0.996 (0.978-1.016)	0.717
			MR Egger	13	1.003 (0.955-1.053)	0.899
			Weighted median	13	1.006 (0.978-1.034)	0.686
			MR-PRESSO†		NA	NA
**MDD**						
PGC	0.545	0.116	**Fixed-effect IVW**	**13**	**0.985 (0.971-0.999)**	**0.033**
			Random effect IVW	13	0.985 (0.972-0.998)	0.025
			Maximum likelihood	13	0.985 (0.971-0.999)	0.031
			MR Egger	13	0.963 (0.934-0.992)	0.030
			Weighted median	13	0.982 (0.963-1.001)	0.063
			MR-PRESSO†		NA	NA
UK Biobank	0.149	0.190	**Fixed-effect IVW**	**13**	**0.878 (0.785-0.982)**	**0.023**
			Random effect IVW	13	0.878 (0.769-1.003)	0.056
			Maximum likelihood	13	0.872 (0.772-0.984)	0.026
			MR Egger	13	1.051 (0.792-1.395)	0.736
			Weighted median	13	0.91 (0.77-1.075)	0.266
			MR-PRESSO†		NA	NA
**BIP**						
PGC	0.008	0.133	Fixed-effect IVW	13	1.013 (0.997-1.029)	0.102
			**Random effect IVW**	**13**	**1.013 (0.99-1.038)**	**0.273**
			Maximum likelihood	13	1.015 (0.998-1.033)	0.090
			MR Egger	13	0.979 (0.934-1.026)	0.395
			Weighted median	13	1.022 (0.997-1.047)	0.090
			MR-PRESSO	12	1.008 (0.992-1.025)	0.333
UK Biobank	0.078	0.301	**Fixed-effect IVW**	**13**	**0.992 (0.926-1.064)**	**0.829**
			Random effect IVW	13	0.992 (0.908-1.084)	0.865
			Maximum likelihood	13	0.992 (0.922-1.067)	0.825
			MR Egger	13	1.092 (0.899-1.327)	0.393

			Weighted median	13	1.015 (0.922-1.118)	0.755
			MR-PRESSO†		NA	NA
FinnGen	0.768	0.578	**Fixed-effect IVW**	**13**	**1.016 (0.982-1.05)**	**0.366**
			Random effect IVW	13	1.016 (0.988-1.044)	0.275
			Maximum likelihood	13	1.016 (0.982-1.051)	0.373
			MR Egger	13	1.034 (0.963-1.111)	0.371
			Weighted median	13	1.025 (0.978-1.074)	0.311
			MR-PRESSO†		NA	NA
**SCZ**						
PGC	0.545	0.695	**Fixed-effect IVW**	**13**	**1.001 (0.987-1.016)**	**0.851**
			Random effect IVW	13	1.001 (0.988-1.015)	0.843
			Maximum likelihood	13	1.001 (0.987-1.017)	0.847
			MR Egger	13	1.007 (0.976-1.039)	0.666
			Weighted median	13	0.998 (0.978-1.018)	0.836
			MR-PRESSO†		NA	NA
UK Biobank	0.330	0.268	**Fixed-effect IVW**	**13**	**0.985 (0.898-1.08)**	**0.744**
			Random effect IVW	13	0.985 (0.893-1.086)	0.759
			Maximum likelihood	13	0.984 (0.895-1.081)	0.736
			MR Egger	13	0.879 (0.709-1.089)	0.262
			Weighted median	13	0.93 (0.818-1.057)	0.266
			MR-PRESSO†		NA	NA
FinnGen	0.858	0.358	**Fixed-effect IVW**	**13**	**1.009 (0.963-1.056)**	**0.711**
			Random effect IVW	13	1.009 (0.974-1.045)	0.627
			Maximum likelihood	13	1.009 (0.963-1.057)	0.710
			MR Egger	13	1.052 (0.954-1.16)	0.330
			Weighted median	13	1.006 (0.945-1.07)	0.859
			MR-PRESSO†		NA	NA
**PTSD**						
PGC	0.521	0.408	**Fixed-effect IVW**	**13**	**1.014 (0.988-1.04)**	**0.299**
			Random effect IVW	13	1.014 (0.989-1.039)	0.280
			Maximum likelihood	13	1.014 (0.988-1.041)	0.308
			MR Egger	13	0.993 (0.94-1.048)	0.793
			Weighted median	13	1 (0.965-1.036)	0.992
			MR-PRESSO†		NA	NA
UK Biobank	0.754	0.749	**Fixed-effect IVW**	**13**	**1.206 (1.009-1.442)**	**0.039**
			Random effect IVW	13	1.206 (1.039-1.4)	0.014
			Maximum likelihood	13	1.21 (1.01-1.449)	0.039
			MR Egger	13	1.279 (0.863-1.898)	0.246
			Weighted median	13	1.276 (1.013-1.609)	0.039
			MR-PRESSO†		NA	NA
FinnGen	0.610	0.369	**Fixed-effect IVW**	**13**	**1.002 (0.945-1.061)**	**0.955**
			Random effect IVW	13	1.002 (0.95-1.056)	0.951
			Maximum likelihood	13	1.002 (0.944-1.063)	0.954
			MR Egger	13	1.054 (0.933-1.191)	0.413
			Weighted median	13	0.973 (0.894-1.058)	0.520
			MR-PRESSO†		NA	NA

**OCD**						
PGC	0.142	0.880	**Fixed-effect IVW**	**13**	**0.974 (0.921-1.031)**	**0.365**
			Random effect IVW	13	0.974 (0.911-1.042)	0.449
			Maximum likelihood	13	0.973 (0.919-1.031)	0.358
			MR Egger	13	0.965 (0.835-1.114)	0.634
			Weighted median	13	0.976 (0.901-1.058)	0.557
			MR-PRESSO†		NA	NA
UK Biobank	0.853	0.355	**Fixed-effect IVW**	**13**	**0.983 (0.824-1.172)**	**0.845**
			Random effect IVW	13	0.983 (0.859-1.125)	0.799
			Maximum likelihood	13	0.982 (0.822-1.174)	0.845
			MR Egger	13	0.828 (0.561-1.222)	0.362
			Weighted median	13	1.01 (0.799-1.279)	0.931
			MR-PRESSO†		NA	NA
FinnGen	0.721	0.194	**Fixed-effect IVW**	**13**	**1.002 (0.942-1.065)**	**0.960**
			Random effect IVW	13	1.002 (0.95-1.056)	0.953
			Maximum likelihood	13	1.002 (0.941-1.066)	0.960
			MR Egger	13	1.085 (0.954-1.234)	0.241
			Weighted median	13	1.013 (0.931-1.102)	0.771
			MR-PRESSO†		NA	NA
**Secondary outcome**						
**Depression**						
PGC & UK Biobank	0.051	0.197	**Fixed-effect IVW**	**12**	**0.997 (0.989-1.005)**	**0.438**
			Random effect IVW	12	0.997 (0.987-1.007)	0.561
			Maximum likelihood	12	0.997 (0.989-1.005)	0.419
			MR Egger	12	0.984 (0.964-1.005)	0.164
			Weighted median	12	0.996 (0.984-1.007)	0.465
			MR-PRESSO†		NA	NA
FinnGen	0.240	0.492	**Fixed-effect IVW**	**13**	**1 (0.986-1.014)**	**0.989**
			Random effect IVW	13	1 (0.985-1.016)	0.990
			Maximum likelihood	13	1 (0.986-1.015)	0.989
			MR Egger	13	0.99 (0.957-1.023)	0.548
			Weighted median	13	0.997 (0.977-1.018)	0.805
			MR-PRESSO†		NA	NA

The best causal estimation highlighted in bold.

AD, anxiety disorders; MDD, major depressive disorder; BIP, bipolar disorder; SCZ, schizophrenia; PTSD, post-traumatic stress disorder; OCD, obsessive-compulsive disorder; PGC, Psychiatric Genomics Consortium; ANGST, Anxiety NeuroGenetics Study; IOCDF-GC, International Obsessive-Compulsive Disorder Foundation Genetics Collaborative; OCGAS, OCD Collaborative Genetics Association Study. P_h, P-value for heterogeneity; P_p, P-value for pleiotropy; NA, not applicable;

*: The ORs are scaled per 0.1 unit increase in log-transformed γ-tocopherol.

†: No significant outliers.

Table S12. The MR analysis results of the causal effects of ascorbate metabolites on major mental disorders.

Outcome	P_h	P_p	MR method	No. SNPs	OR (95% CI)	*P*-value
**Primary outcome**						
**AD**						
ANGST Consortium	0.731	0.609	**Fixed-effect IVW**	**13**	**1.225 (0.965-1.556)**	**0.095**
			Random effect IVW	13	1.225 (1-1.501)	0.050
			Maximum likelihood	13	1.232 (0.963-1.578)	0.097
			MR Egger	13	1.095 (0.678-1.771)	0.717
			Weighted median	13	1.197 (0.866-1.655)	0.276
			MR-PRESSO†		NA	NA
UK Biobank	0.592	0.969	**Fixed-effect IVW**	**14**	**1.029 (0.94-1.126)**	**0.533**
			Random effect IVW	14	1.029 (0.947-1.119)	0.502
			Maximum likelihood	14	1.03 (0.941-1.127)	0.527
			MR Egger	14	1.032 (0.881-1.208)	0.704
			Weighted median	14	1.038 (0.918-1.174)	0.550
			MR-PRESSO†		NA	NA
FinnGen	0.370	0.461	**Fixed-effect IVW**	**14**	**1.014 (0.942-1.091)**	**0.714**
			Random effect IVW	14	1.014 (0.939-1.094)	0.724
			Maximum likelihood	14	1.014 (0.941-1.093)	0.711
			MR Egger	14	0.972 (0.85-1.111)	0.682
			Weighted median	14	1.014 (0.92-1.119)	0.774
			MR-PRESSO†		NA	NA
**MDD**						
PGC	0.348	0.913	**Fixed-effect IVW**	**14**	**1.061 (1-1.126)**	**0.050**
			Random effect IVW	14	1.061 (0.997-1.13)	0.062
			Maximum likelihood	14	1.063 (1.001-1.13)	0.047
			MR Egger	14	1.055 (0.933-1.194)	0.411
			Weighted median	14	1.079 (0.995-1.17)	0.066
			MR-PRESSO†		NA	NA
UK Biobank	0.121	0.623	**Fixed-effect IVW**	**14**	**0.657 (0.442-0.976)**	**0.037**
			Random effect IVW	14	0.657 (0.407-1.061)	0.086
			Maximum likelihood	14	0.657 (0.435-0.992)	0.046
			MR Egger	14	0.789 (0.332-1.872)	0.601
			Weighted median	14	0.555 (0.295-1.044)	0.068
			MR-PRESSO†		NA	NA
**BIP**						
PGC	0.138	0.288	**Fixed-effect IVW**	**14**	**1.037 (0.974-1.105)**	**0.258**
			Random effect IVW	14	1.037 (0.962-1.119)	0.343
			Maximum likelihood	14	1.039 (0.974-1.109)	0.241
			MR Egger	14	1.105 (0.966-1.264)	0.171
			Weighted median	14	1.05 (0.956-1.152)	0.307
			MR-PRESSO†		NA	NA
UK Biobank	0.927	0.742	**Fixed-effect IVW**	**14**	**0.883 (0.69-1.129)**	**0.320**
			Random effect IVW	14	0.883 (0.742-1.05)	0.159
			Maximum likelihood	14	0.885 (0.689-1.137)	0.339
			MR Egger	14	0.938 (0.609-1.444)	0.776

			Weighted median	14	0.923 (0.643-1.325)	0.664
			MR-PRESSO†		NA	NA
FinnGen	0.847	0.881	**Fixed-effect IVW**	**14**	**1.045 (0.914-1.196)**	**0.517**
			Random effect IVW	14	1.045 (0.941-1.161)	0.407
			Maximum likelihood	14	1.047 (0.914-1.201)	0.507
			MR Egger	14	1.061 (0.841-1.338)	0.626
			Weighted median	14	1.026 (0.827-1.273)	0.813
			MR-PRESSO†		NA	NA
**SCZ**						
PGC	0.348	0.450	**Fixed-effect IVW**	**14**	**1.004 (0.948-1.064)**	**0.883**
			Random effect IVW	14	1.004 (0.945-1.068)	0.889
			Maximum likelihood	14	1.005 (0.947-1.066)	0.881
			MR Egger	14	1.042 (0.932-1.164)	0.483
			Weighted median	14	1.008 (0.928-1.095)	0.845
			MR-PRESSO†		NA	NA
UK Biobank	0.485	0.919	**Fixed-effect IVW**	**14**	**0.812 (0.585-1.125)**	**0.210**
			Random effect IVW	14	0.812 (0.589-1.118)	0.202
			Maximum likelihood	14	0.814 (0.585-1.135)	0.225
			MR Egger	14	0.833 (0.464-1.493)	0.550
			Weighted median	14	0.788 (0.507-1.223)	0.288
			MR-PRESSO†		NA	NA
FinnGen	0.616	0.604	**Fixed-effect IVW**	**14**	**1.121 (0.931-1.348)**	**0.227**
			Random effect IVW	14	1.121 (0.946-1.328)	0.188
			Maximum likelihood	14	1.127 (0.926-1.372)	0.234
			MR Egger	14	1.044 (0.759-1.437)	0.796
			Weighted median	14	1.09 (0.842-1.41)	0.512
			MR-PRESSO†		NA	NA
**PTSD**						
PGC	0.210	0.227	**Fixed-effect IVW**	**14**	**0.972 (0.878-1.076)**	**0.588**
			Random effect IVW	14	0.972 (0.866-1.091)	0.633
			Maximum likelihood	14	0.971 (0.875-1.076)	0.571
			MR Egger	14	0.871 (0.71-1.068)	0.208
			Weighted median	14	0.955 (0.825-1.105)	0.536
			MR-PRESSO†		NA	NA
UK Biobank	0.240	0.375	**Fixed-effect IVW**	**14**	**0.814 (0.434-1.529)**	**0.523**
			Random effect IVW	14	0.814 (0.403-1.644)	0.567
			Maximum likelihood	14	0.818 (0.434-1.541)	0.534
			MR Egger	14	0.505 (0.147-1.742)	0.301
			Weighted median	14	0.649 (0.275-1.529)	0.323
			MR-PRESSO†		NA	NA
FinnGen	0.089	0.997	**Fixed-effect IVW**	**14**	**1.166 (0.927-1.469)**	**0.190**
			Random effect IVW	14	1.166 (0.875-1.555)	0.294
			Maximum likelihood	14	1.173 (0.927-1.484)	0.183
			MR Egger	14	1.168 (0.696-1.958)	0.568
			Weighted median	14	1.189 (0.86-1.643)	0.295
			MR-PRESSO†		NA	NA

**OCD**						
IOCDF-GC & OCGAS	0.270	0.047	Fixed-effect IVW	14	1.435 (1.121-1.837)	0.004
			Random effect IVW	14	1.435 (1.095-1.882)	0.009
			Maximum likelihood	14	1.492 (1.121-1.986)	0.006
			**MR Egger**	**14**	**0.906 (0.563-1.459)**	**0.692**
			Weighted median	14	1.153 (0.803-1.655)	0.441
			MR-PRESSO†		NA	NA
UK Biobank	0.223	0.579	**Fixed-effect IVW**	**14**	**1.555 (0.835-2.897)**	**0.164**
			Random effect IVW	14	1.555 (0.772-3.134)	0.217
			Maximum likelihood	14	1.566 (0.844-2.906)	0.155
			MR Egger	14	2.102 (0.596-7.411)	0.271
			Weighted median	14	1.78 (0.7-4.53)	0.226
			MR-PRESSO†		NA	NA
FinnGen	0.600	0.679	**Fixed-effect IVW**	**14**	**1.071 (0.836-1.372)**	**0.588**
			Random effect IVW	14	1.071 (0.852-1.346)	0.558
			Maximum likelihood	14	1.075 (0.831-1.39)	0.582
			MR Egger	14	0.993 (0.648-1.523)	0.975
			Weighted median	14	0.99 (0.699-1.403)	0.955
			MR-PRESSO†		NA	NA
**Secondary outcome**						
**Depression**						
PGC & UK Biobank	0.041	0.084	Fixed-effect IVW	14	1.003 (0.976-1.031)	0.832
			**Random effect IVW**	**14**	**1.003 (0.967-1.04)**	**0.873**
			Maximum likelihood	14	1.003 (0.974-1.033)	0.822
			MR Egger	14	0.957 (0.902-1.015)	0.171
			Weighted median	14	1.003 (0.96-1.049)	0.882
			MR-PRESSO†		NA	NA
FinnGen	0.784	0.750	**Fixed-effect IVW**	**14**	**1.057 (1-1.117)**	**0.050**
			Random effect IVW	14	1.057 (1.01-1.106)	0.018
			Maximum likelihood	14	1.059 (0.998-1.123)	0.056
			MR Egger	14	1.043 (0.948-1.148)	0.402
			Weighted median	14	1.043 (0.966-1.126)	0.277
			MR-PRESSO†		NA	NA

The best causal estimation highlighted in bold.

AD, anxiety disorders; MDD, major depressive disorder; BIP, bipolar disorder; SCZ, schizophrenia; PTSD, post-traumatic stress disorder; OCD, obsessive-compulsive disorder; PGC, Psychiatric Genomics Consortium; ANGST, Anxiety NeuroGenetics Study; IOCDF-GC, International Obsessive-Compulsive Disorder Foundation Genetics Collaborative; OCGAS, OCD Collaborative Genetics Association Study. P_h, P-value for heterogeneity; P_p, P-value for pleiotropy; NA, not applicable;

*: The ORs are scaled per unit increase in log-transformed ascorbate.

†: No significant outliers.

Table S13. The MR analysis results of the causal effects of retinol metabolites on major mental disorders.

Outcome	P_h	P_p	MR method	No. SNPs	OR (95% CI)	*P*-value
**Primary outcome**						
**AD**						
ANGST Consortium	<0.001	0.971	Fixed-effect IVW	20	1.014 (0.937-1.097)	0.730
			**Random effect IVW**	**20**	**1.014 (0.897-1.147)**	**0.825**
			Maximum likelihood	20	1.016 (0.932-1.107)	0.714
			MR Egger	20	1.005 (0.621-1.628)	0.984
			Weighted median	20	1.018 (0.897-1.154)	0.786
			MR-PRESSO	19	0.989 (0.889-1.099)	0.832
UK Biobank	0.376	0.320	**Fixed-effect IVW**	**24**	**0.993 (0.959-1.028)**	**0.699**
			Random effect IVW	24	0.993 (0.958-1.029)	0.708
			Maximum likelihood	24	0.993 (0.958-1.029)	0.689
			MR Egger	24	0.95 (0.865-1.043)	0.290
			Weighted median	24	0.97 (0.923-1.019)	0.224
			MR-PRESSO†		NA	NA
FinnGen	0.058	0.617	**Fixed-effect IVW**	**25**	**0.972 (0.946-0.998)**	**0.037**
			Random effect IVW	25	0.972 (0.941-1.004)	0.087
			Maximum likelihood	25	0.971 (0.944-0.999)	0.044
			MR Egger	25	0.992 (0.911-1.079)	0.848
			Weighted median	25	0.99 (0.951-1.031)	0.633
			MR-PRESSO†		NA	NA
**MDD**						
PGC	0.583	0.877	**Fixed-effect IVW**	**25**	**0.982 (0.963-1.001)**	**0.067**
			Random effect IVW	25	0.982 (0.964-1)	0.056
			Maximum likelihood	25	0.982 (0.962-1.002)	0.074
			MR Egger	25	0.986 (0.936-1.038)	0.597
			Weighted median	25	0.992 (0.964-1.021)	0.570
			MR-PRESSO†		NA	NA
UK Biobank	0.015	0.573	Fixed-effect IVW	24	0.901 (0.774-1.049)	0.179
			**Random effect IVW**	**24**	**0.901 (0.737-1.101)**	**0.309**
			Maximum likelihood	24	0.899 (0.764-1.058)	0.200
			MR Egger	24	1.041 (0.61-1.774)	0.885
			Weighted median	24	0.904 (0.712-1.148)	0.407
			MR-PRESSO†		NA	NA
**BIP**						
PGC	0.417	0.351	**Fixed-effect IVW**	**24**	**0.992 (0.97-1.015)**	**0.484**
			Random effect IVW	24	0.992 (0.969-1.015)	0.491
			Maximum likelihood	24	0.992 (0.969-1.015)	0.478
			MR Egger	24	1.02 (0.959-1.085)	0.536
			Weighted median	24	1.003 (0.971-1.036)	0.846
			MR-PRESSO†		NA	NA
UK Biobank	0.736	0.130	**Fixed-effect IVW**	**24**	**1.046 (0.951-1.15)**	**0.354**
			Random effect IVW	24	1.046 (0.961-1.138)	0.300
			Maximum likelihood	24	1.048 (0.951-1.155)	0.345
			MR Egger	24	1.257 (0.981-1.61)	0.084

			Weighted median	24	1.007 (0.884-1.148)	0.912
			MR-PRESSO†		NA	NA
FinnGen	0.743	0.209	**Fixed-effect IVW**	**25**	**0.943 (0.898-0.991)**	**0.020**
			Random effect IVW	25	0.943 (0.903-0.986)	0.009
			Maximum likelihood	25	0.942 (0.896-0.991)	0.021
			MR Egger	25	1.018 (0.898-1.154)	0.787
			Weighted median	25	0.938 (0.873-1.008)	0.081
			MR-PRESSO†		NA	NA
**SCZ**						
PGC	0.003	0.799	Fixed-effect IVW	24	0.994 (0.973-1.015)	0.561
			**Random effect IVW**	**24**	**0.994 (0.965-1.024)**	**0.682**
			Maximum likelihood	24	0.993 (0.972-1.016)	0.561
			MR Egger	24	1.004 (0.925-1.089)	0.928
			Weighted median	24	1.014 (0.982-1.047)	0.400
			MR-PRESSO	22	1.007 (0.985-1.029)	0.546
UK Biobank	0.309	0.408	**Fixed-effect IVW**	**24**	**0.975 (0.86-1.105)**	**0.689**
			Random effect IVW	24	0.975 (0.853-1.113)	0.706
			Maximum likelihood	24	0.973 (0.854-1.109)	0.682
			MR Egger	24	1.12 (0.79-1.589)	0.532
			Weighted median	24	1.086 (0.902-1.308)	0.385
			MR-PRESSO†		NA	NA
FinnGen	0.785	0.758	**Fixed-effect IVW**	**25**	**0.978 (0.915-1.047)**	**0.526**
			Random effect IVW	25	0.978 (0.923-1.038)	0.469
			Maximum likelihood	25	0.978 (0.913-1.048)	0.530
			MR Egger	25	0.954 (0.804-1.132)	0.597
			Weighted median	25	1.009 (0.915-1.114)	0.854
			MR-PRESSO†		NA	NA
**PTSD**						
PGC	0.400	1 0.6271677	**Fixed-effect IVW**	**23**	**0.994 (0.958-1.032)**	**0.760**
			Random effect IVW	23	0.994 (0.957-1.033)	0.765
			Maximum likelihood	23	0.994 (0.956-1.033)	0.754
			MR Egger	23	0.969 (0.868-1.081)	0.578
			Weighted median	23	1.01 (0.957-1.067)	0.712
			MR-PRESSO†		NA	NA
UK Biobank	0.661	0.751	**Fixed-effect IVW**	**24**	**0.906 (0.711-1.154)**	**0.424**
			Random effect IVW	24	0.906 (0.724-1.134)	0.388
			Maximum likelihood	24	0.904 (0.704-1.16)	0.428
			MR Egger	24	0.997 (0.529-1.879)	0.993
			Weighted median	24	0.88 (0.633-1.224)	0.449
			MR-PRESSO†		NA	NA
FinnGen	0.417	0.783	**Fixed-effect IVW**	**25**	**0.962 (0.884-1.047)**	**0.370**
			Random effect IVW	25	0.962 (0.883-1.048)	0.378
			Maximum likelihood	25	0.961 (0.881-1.048)	0.369
			MR Egger	25	0.935 (0.748-1.167)	0.557
			Weighted median	25	0.997 (0.882-1.128)	0.962
			MR-PRESSO†		NA	NA

**OCD**						
IOCDF-GC & OCGAS	0.456	0.721	**Fixed-effect IVW**	**24**	**0.984 (0.905-1.07)**	**0.706**
			Random effect IVW	24	0.984 (0.905-1.07)	0.706
			Maximum likelihood	24	0.983 (0.901-1.072)	0.691
			MR Egger	24	0.945 (0.745-1.198)	0.643
			Weighted median	24	0.98 (0.868-1.106)	0.742
			MR-PRESSO†		NA	NA
UK Biobank	0.373	0.377	**Fixed-effect IVW**	**24**	**0.955 (0.752-1.213)**	**0.707**
			Random effect IVW	24	0.955 (0.746-1.223)	0.716
			Maximum likelihood	24	0.954 (0.745-1.222)	0.710
			MR Egger	24	1.259 (0.658-2.409)	0.494
			Weighted median	24	0.974 (0.687-1.383)	0.884
			MR-PRESSO†		NA	NA
FinnGen	0.334	0.267	**Fixed-effect IVW**	**25**	**0.919 (0.84-1.006)**	**0.068**
			Random effect IVW	25	0.919 (0.836-1.011)	0.081
			Maximum likelihood	25	0.916 (0.834-1.007)	0.069
			MR Egger	25	1.045 (0.822-1.327)	0.724
			Weighted median	25	0.936 (0.824-1.063)	0.311
			MR-PRESSO†		NA	NA
**Secondary outcome**						
**Depression**						
PGC & UK Biobank	0.071	0.599	**Fixed-effect IVW**	**24**	**0.994 (0.984-1.005)**	**0.285**
			Random effect IVW	24	0.994 (0.982-1.007)	0.376
			Maximum likelihood	24	0.994 (0.984-1.005)	0.292
			MR Egger	24	1.003 (0.97-1.036)	0.872
			Weighted median	24	0.995 (0.98-1.01)	0.484
			MR-PRESSO†		NA	NA
FinnGen	0.051	0.309	**Fixed-effect IVW**	**25**	**0.971 (0.951-0.991)**	**0.004**
			Random effect IVW	25	0.971 (0.947-0.995)	0.019
			Maximum likelihood	25	0.97 (0.949-0.991)	0.005
			MR Egger	25	1.001 (0.94-1.067)	0.971
			Weighted median	25	0.984 (0.953-1.017)	0.347
			MR-PRESSO†		NA	NA

The best causal estimation highlighted in bold.

AD, anxiety disorders; MDD, major depressive disorder; BIP, bipolar disorder; SCZ, schizophrenia; PTSD, post-traumatic stress disorder; OCD, obsessive-compulsive disorder; PGC, Psychiatric Genomics Consortium; ANGST, Anxiety NeuroGenetics Study; IOCDF-GC, International Obsessive-Compulsive Disorder Foundation Genetics Collaborative; OCGAS, OCD Collaborative Genetics Association Study. P_h, *P*-value for heterogeneity; P_p, *P*-value for pleiotropy; NA, not applicable;

*: The ORs are scaled per unit increase in log-transformed retinol.

†: No significant outliers.

References:

1. Major, J.M.; Yu, K.; Wheeler, W.; Zhang, H.; Cornelis, M.C.; Wright, M.E.; Yeager, M.; Snyder, K.; Weinstein, S.J.; Mondul, A.; et al. Genome-wide association study identifies common variants associated with circulating vitamin E levels. *Hum Mol Genet* **2011**, 20, 3876-3883.
2. Zheng, J.S.; Luan, J.; Sofianopoulou, E.; Imamura, F.; Stewart, I.D.; Day, F.R.; Pietzner, M.; Wheeler, E.; Lotta, L.A.; Gundersen, T.E.; et al. Plasma Vitamin C and Type 2 Diabetes: Genome-Wide Association Study and Mendelian Randomization Analysis in European Populations. *Diabetes Care* **2021**, 44, 98-106.
3. Mondul, A.M.; Yu, K.; Wheeler, W.; Zhang, H.; Weinstein, S.J.; Major, J.M.; Cornelis, M.C.; Männistö, S.; Hazra, A.; Hsing, A.W.; et al. Genome-wide association study of circulating retinol levels. *Hum Mol Genet* **2011**, 20, 4724-4731.
4. Hendrickson, S.J.; Hazra, A.; Chen, C.; Eliassen, A.H.; Kraft, P.; Rosner, B.A.; Willett, W.C. β-Carotene 15,15'-monooxygenase 1 single nucleotide polymorphisms in relation to plasma carotenoid and retinol concentrations in women of European descent. *Am J Clin Nutr* **2012**, 96, 1379-1389.
5. D'Adamo, C.R.; D'Urso, A.; Ryan, K.A.; Yerges-Armstrong, L.M.; Semba, R.D.; Steinle, N.I.; Mitchell, B.D.; Shuldiner, A.R.; McArdle, P.F. A Common Variant in the SETD7 Gene Predicts Serum Lycopene Concentrations. *Nutrients* **2016**, 8, 82.
6. Otowa, T.; Hek, K.; Lee, M.; Byrne, E.M.; Mirza, S.S.; Nivard, M.G.; Bigdeli, T.; Aggen, S.H.; Adkins, D.; Wolen, A.; et al. Meta-analysis of genome-wide association studies of anxiety disorders. *Mol Psychiatry* **2016**, 21, 1391-1399.
7. Kurki, M.I.; Karjalainen, J.; Palta, P.; Sipilä, T.P.; Kristiansson, K.; Donner, K.; Reeve, M.P.; Laivuori, H.; Aavikko, M.; Kaunisto, M.A.; et al. FinnGen: Unique genetic insights from combining isolated population and national health register data. *medRxiv* **2022**, 2022.03.03.22271360.
8. Wray, N.R.; Ripke, S.; Mattheisen, M.; Trzaskowski, M.; Byrne, E.M.; Abdellaoui, A.; Adams, M.J.; Agerbo, E.; Air, T.M.; Andlauer, T.M.F.; et al. Genome-wide association analyses identify 44 risk variants and refine the genetic architecture of major depression. *Nat Genet* **2018**, 50, 668-681.
9. Mullins, N.; Forstner, A.J.; O'Connell, K.S.; Coombes, B.; Coleman, J.R.I.; Qiao, Z.; Als, T.D.; Bigdeli, T.B.; Børte, S.; Bryois, J.; et al. Genome-wide association study of more than 40,000 bipolar disorder cases provides new insights into the underlying biology. *Nat Genet* **2021**, 53, 817-829.
10. Trubetskoy, V.; Pardiñas, A.F.; Qi, T.; Panagiotaropoulou, G.; Awasthi, S.; Bigdeli, T.B.; Bryois, J.; Chen, C.Y.; Dennison, C.A.; Hall, L.S.; et al. Mapping genomic loci implicates genes and synaptic biology in schizophrenia. *Nature* **2022**, 604, 502-508.
11. Nievergelt, C.M.; Maihofer, A.X.; Klengel, T.; Atkinson, E.G.; Chen, C.Y.; Choi, K.W.; Coleman, J.R.I.; Dalvie, S.; Duncan, L.E.; Gelernter, J.; et al. International meta-analysis of PTSD genome-wide association studies identifies sex- and ancestry-specific genetic risk loci. *Nat Commun* **2019**, 10, 4558.
12. International Obsessive Compulsive Disorder Foundation Genetics Collaborative (IOCDF-GC) and OCD Collaborative Genetics Association Studies (OCGAS). Revealing the complex genetic architecture of obsessive-compulsive disorder using meta-analysis. *Mol Psychiatry* **2018**, 23, 1181-1188.
13. Howard, D.M.; Adams, M.J.; Clarke, T.K.; Hafferty, J.D.; Gibson, J.; Shirali, M.; Coleman, J.R.I.; Hagenaars, S.P.; Ward, J.; Wigmore, E.M.; et al. Genome-wide meta-analysis of depression identifies 102 independent variants and highlights the importance of the prefrontal brain regions. *Nat Neurosci* **2019**, 22, 343-352.
